# Supplementary material for: A PCR primer design method for identifying spider mite species using k-mer counting
Source: PLoS One. 2025 Jun 9;20(6):e0321199. doi: 10.1371/journal.pone.0321199 (PMC12148119; doi:10.1371/journal.pone.0321199)

(a) Agarose gel showing results of PCR amplification using primers using Pci6_F1 and Pci6_R1 showing the *P. citri* (lane 1) and *P. osmanthi* (lane 4) bands.

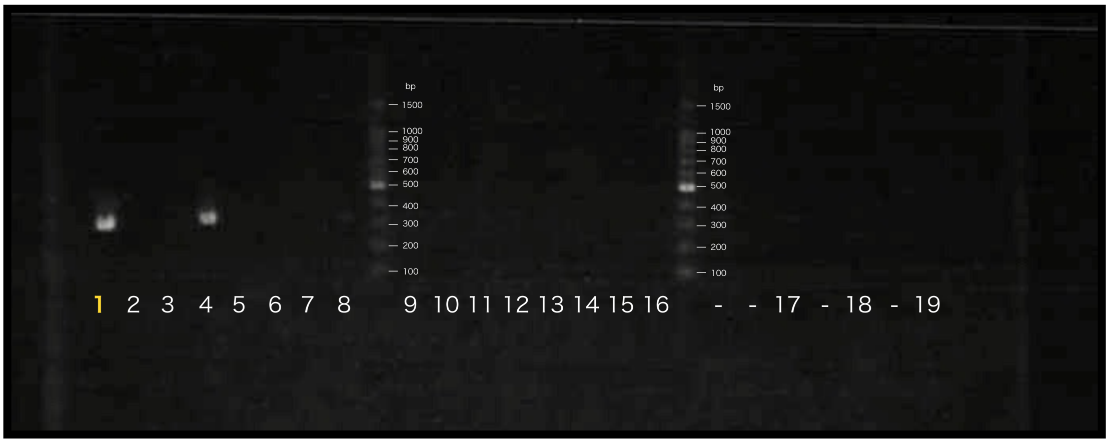

(b) Agarose gel showing results of PCR amplification using primers using Pmo1_F2 and Pmo1_R2 showing the *P. mori* (lane 2) band.

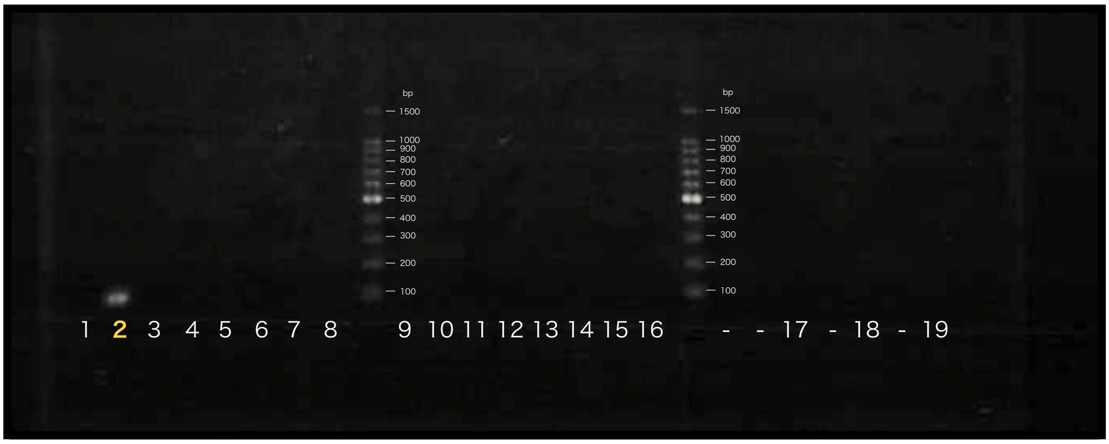

(c) Agarose gel showing results of PCR amplification using primers using Pul7_F1 and Pul7_R1 showing the *P. ulmi* (lane 3) band.

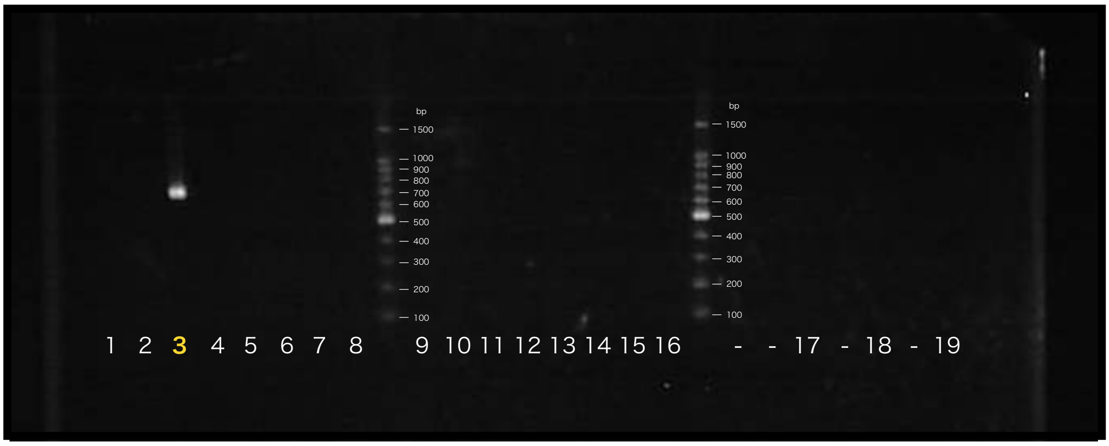

(d) Agarose gel showing results of PCR amplification using primers using Pos5_F1 and Pos5_R1 showing the *P. osmanthi* (lane 4) band.

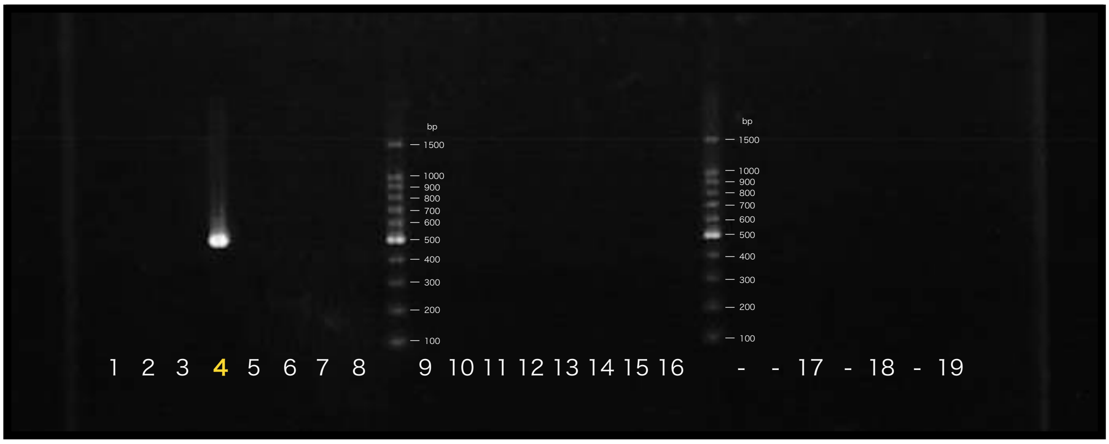

(e) Agarose gel showing results of PCR amplification using primers using Ssh7_F1 and Ssh7_R1 showing the *S. shii* (lane 5) band.

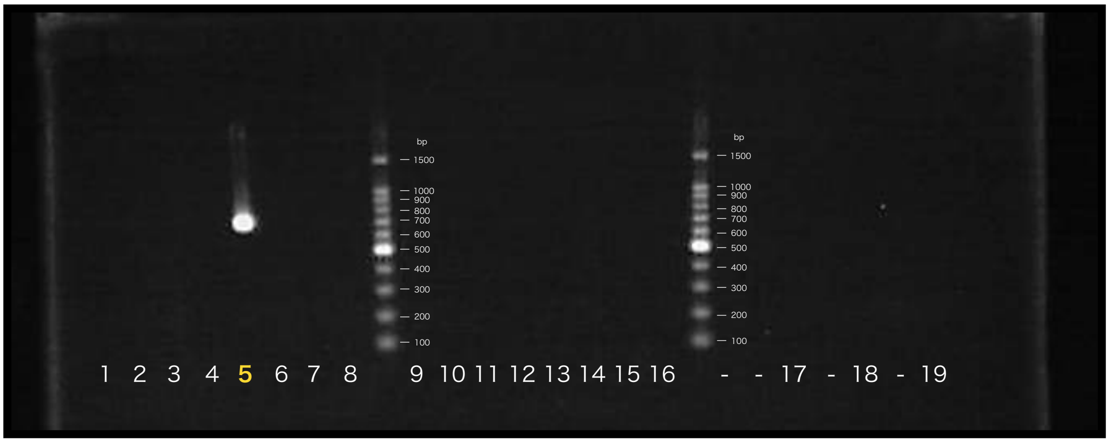

(f) Agarose gel showing results of PCR amplification using primers using Eno6_F1 and Eno6_R1 showing the *E. nomurai* (lane 6) band.

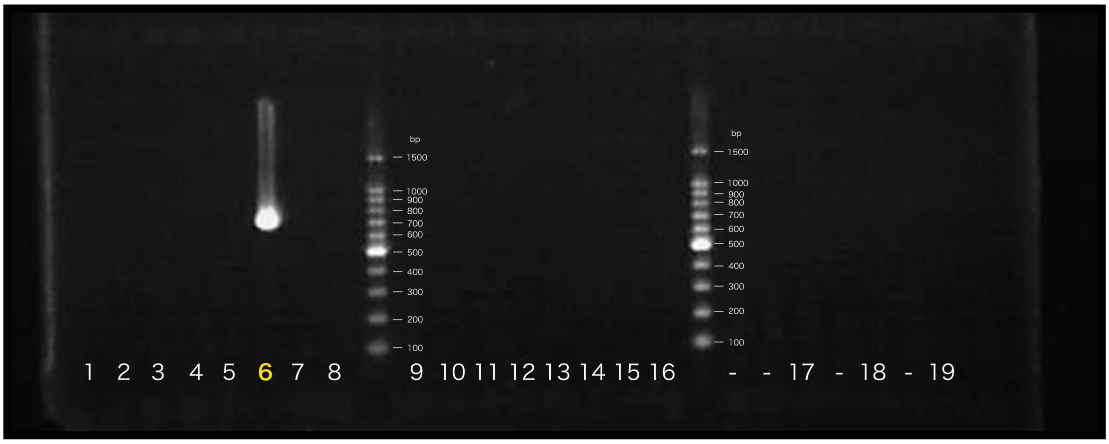

(g) Agarose gel showing results of PCR amplification using primers using Ece3_F1 and Ece3_R1 showing the *E. celtis* (lane 7) band.

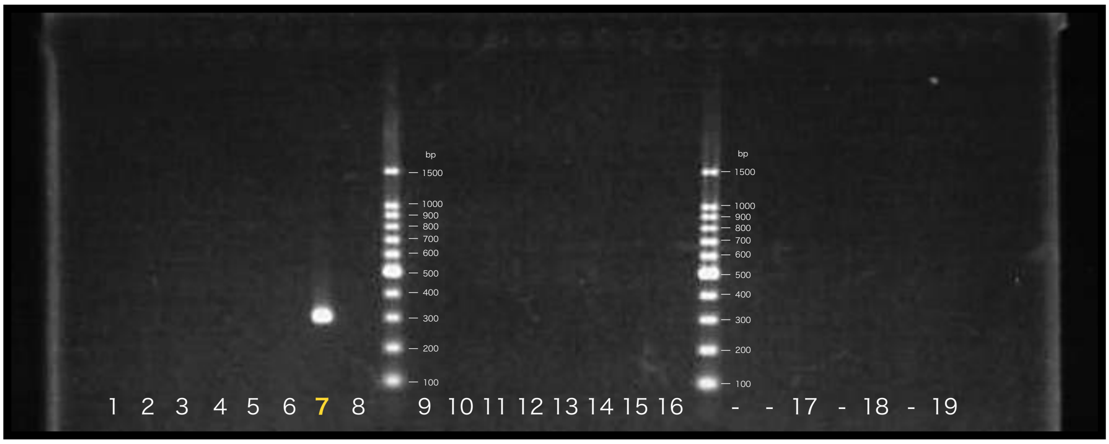

(h) Agarose gel showing results of PCR amplification using primers using Ece5_F2 and Ece5_R2 showing the *E. celtis* (lane 7) band.

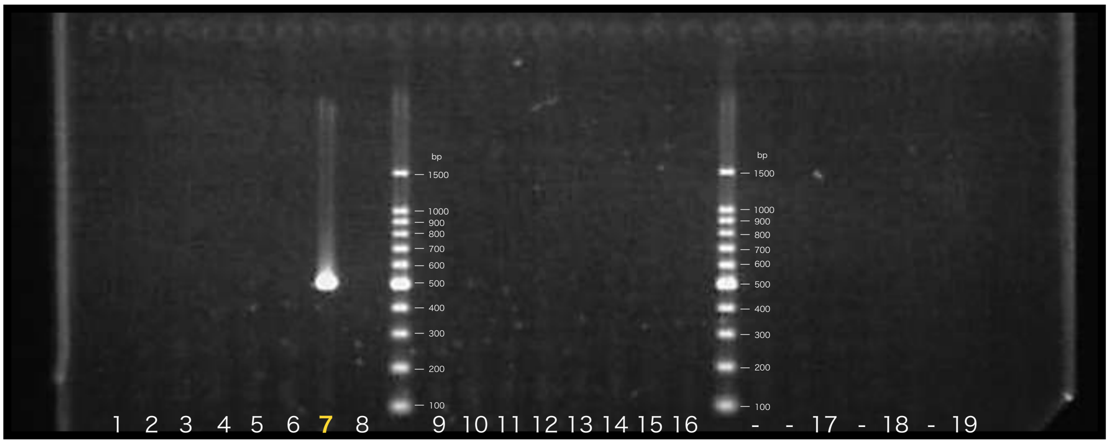

(i) Agarose gel showing results of PCR amplification using primers using Oca4_F1 and Oca4_R1 showing the *O. castaneae* (lane 8) band.

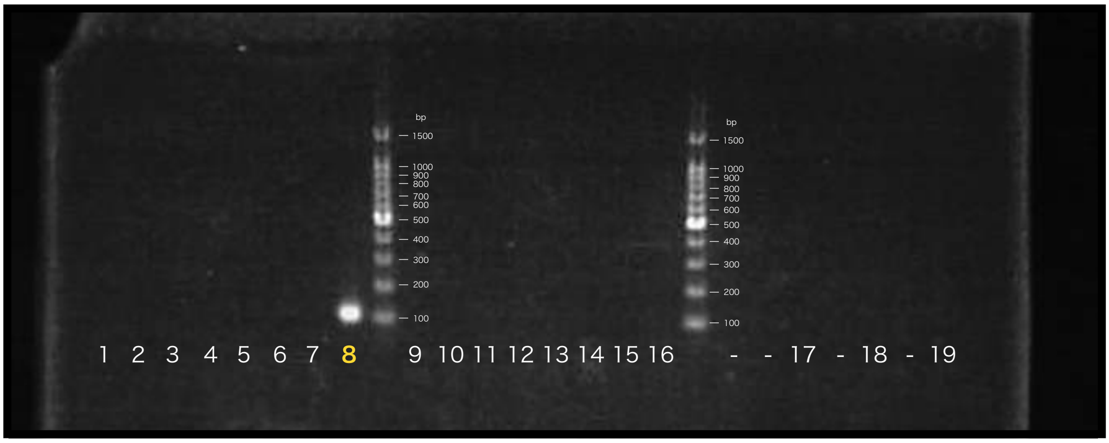

(j) Agarose gel showing results of PCR amplification using primers using Oca1_F1 and Oca1_R1 showing the *O. castaneae* (lane 8) band.

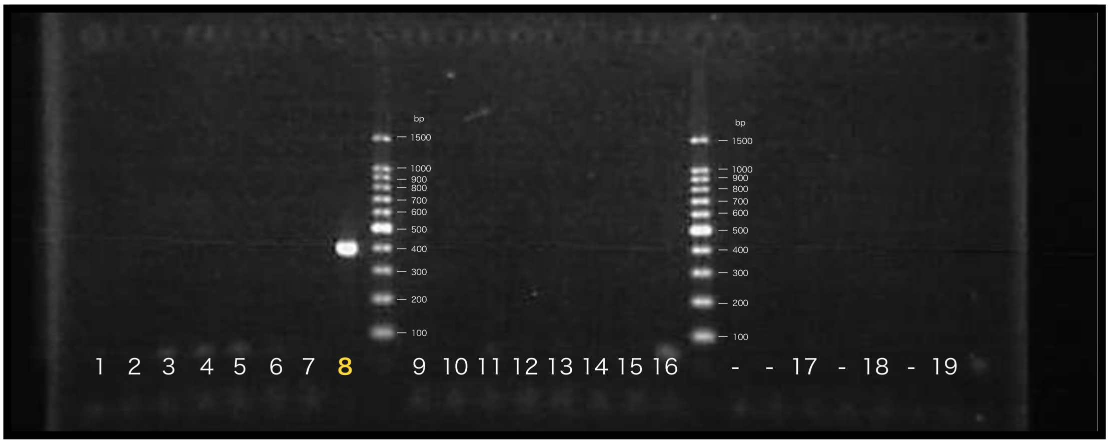

(k) Agarose gel showing results of PCR amplification using primers using Oil2_F1 and Oil2_R1 showing the *O. ilicis* (lane 9) band.

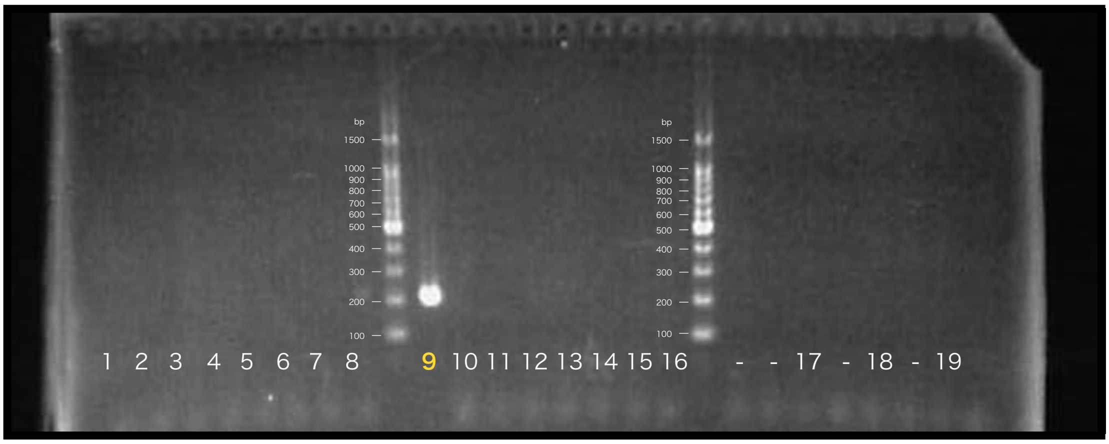

(l) Agarose gel showing results of PCR amplification using primers using Oco1_F1 and Oco1_R1 showing the *O. coffeae* (lane 10) band.

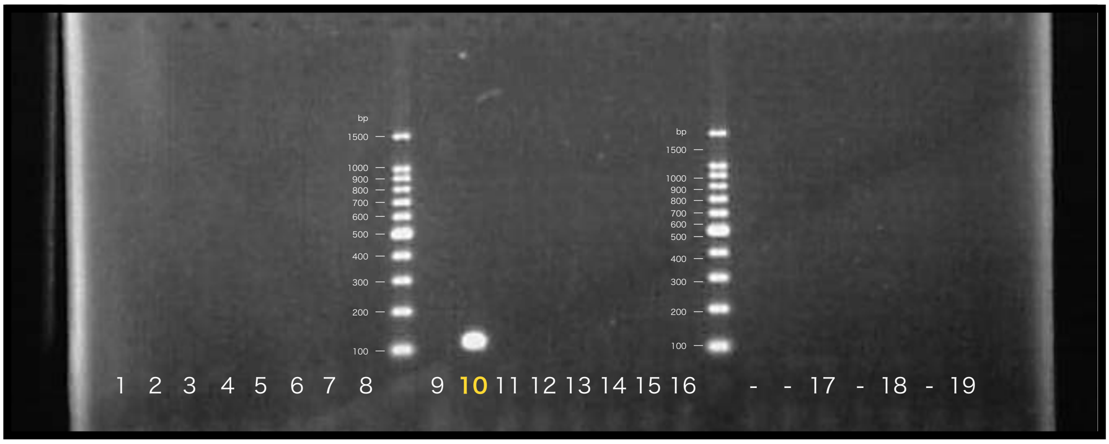

(m) Agarose gel showing results of PCR amplification using primers using Ogo7_F1 and Ogo7_R1 showing the *O. gotohi* (lane 11) band.

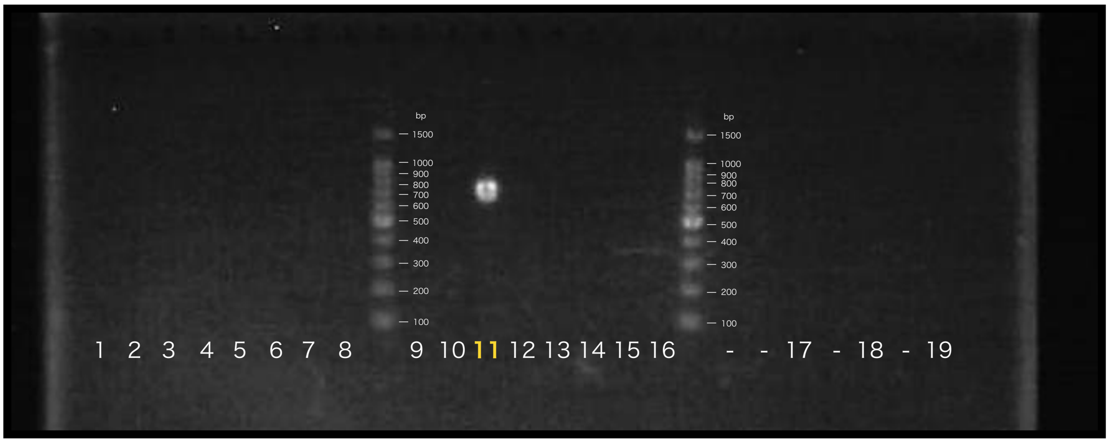

(n) Agarose gel showing results of PCR amplification using primers using Oam2_F1 and Oam2_R1 showing the *O. amiensis* (lane 12) band.

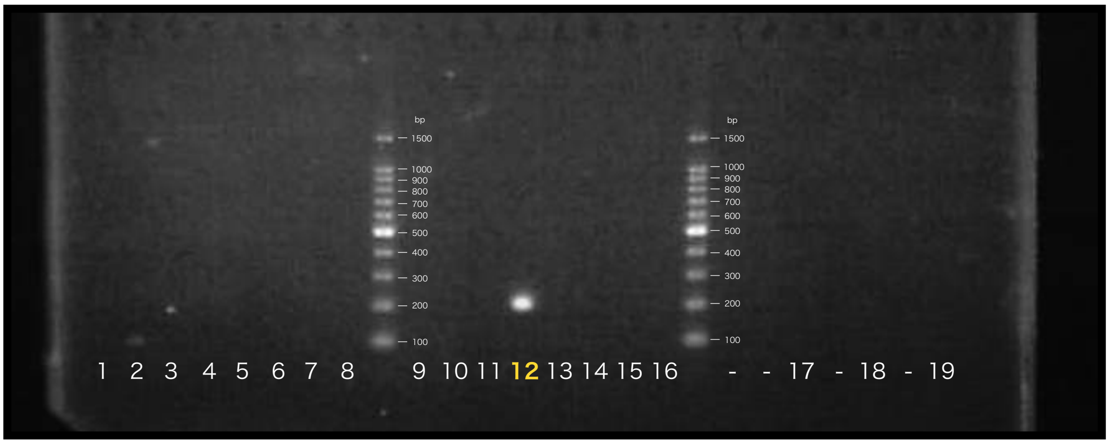

(o) Agarose gel showing results of PCR amplification using primers using Tka8_F1 and Tka8_R1 showing the *T. kanzawai* (lane 13) band.

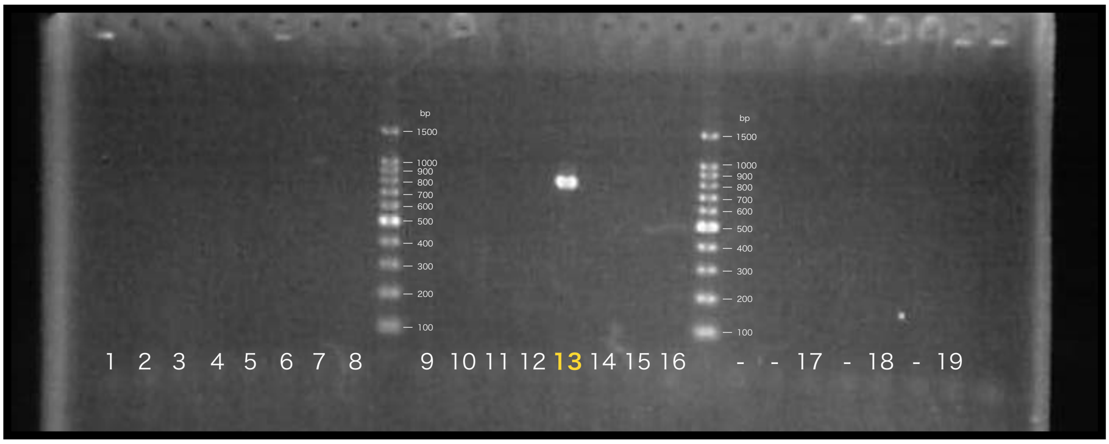

(p) Agarose gel showing results of PCR amplification using primers using Tpar2_F1 and Tpar2_R1 showing the *T. parakanzawai* (lane 14) band.

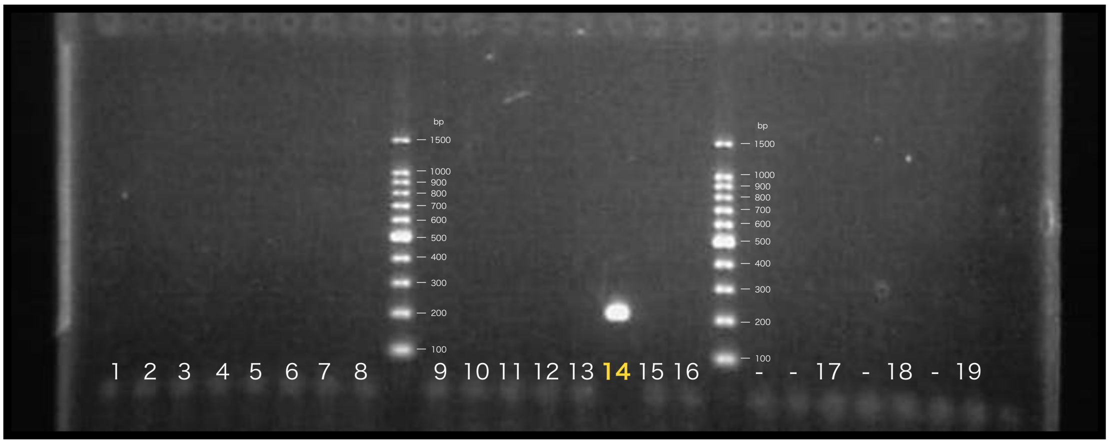

(q) Agarose gel showing results of PCR amplification using primers using TurR6_F1 and TurR6_R1 showing the *T. urticae* (red_form, lane 15) band.

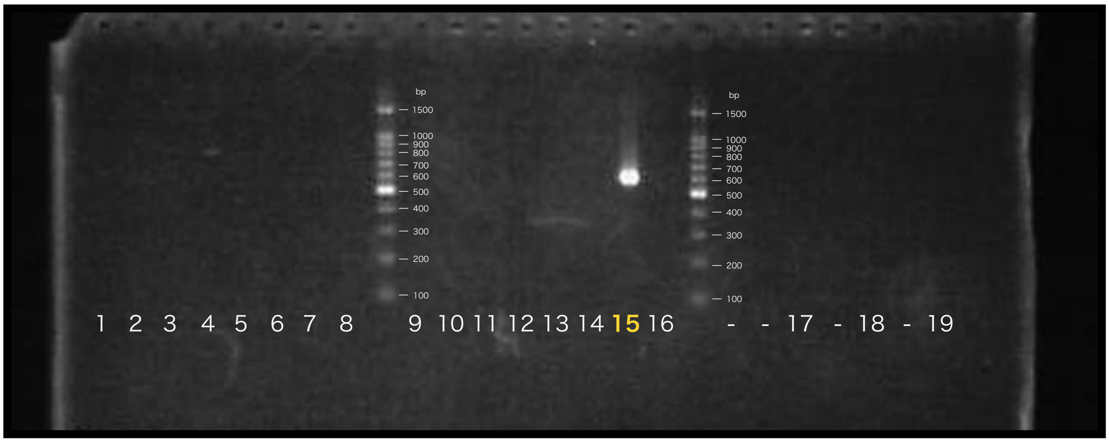

(r) Agarose gel showing results of PCR amplification using primers using TurG5_F2 and TurG5_R2 showing non-amplification (lane 16).

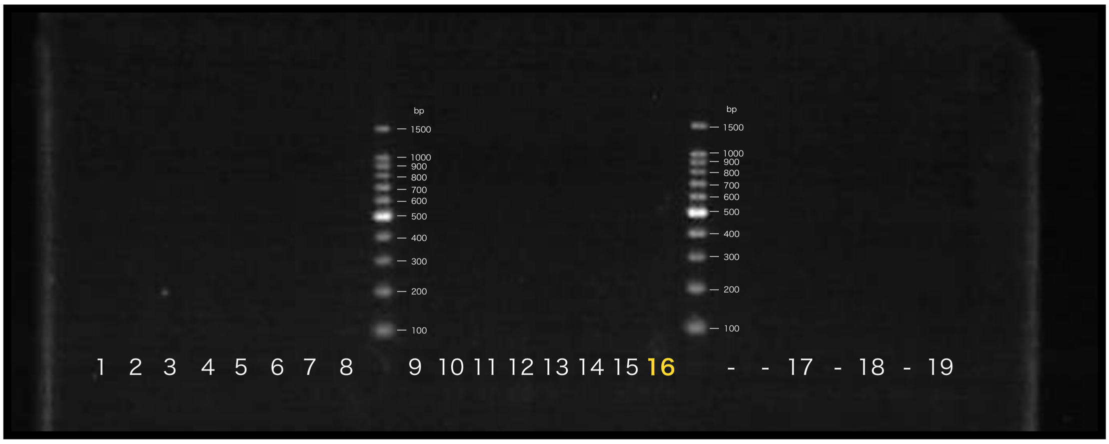

(s) Agarose gel showing results of PCR amplification using primers using Ttr4_F1 and Ttr4_R1 showing the *T. truncatus* (lane 17) band.

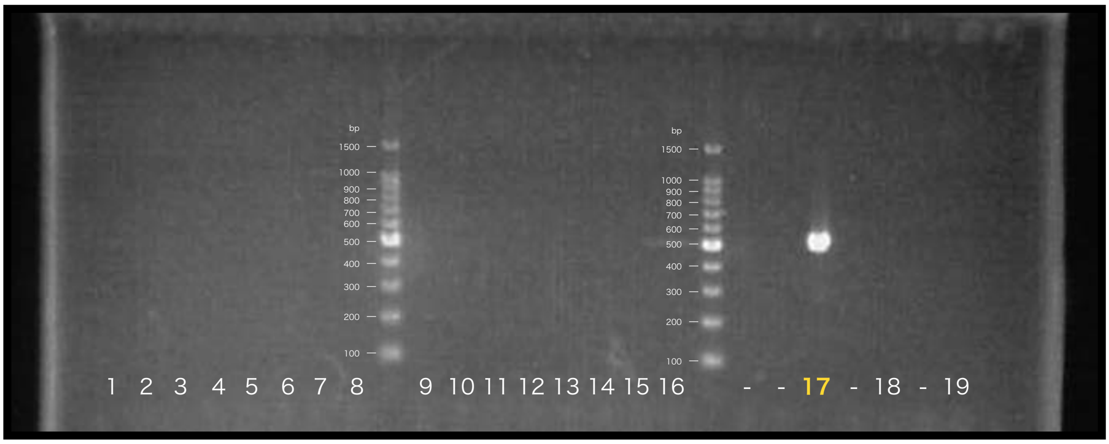

(t) Agarose gel showing results of PCR amplification using primers using Tpu6_F1 and Tpu6_R1 showing the *T. puerariicola* (lane 18) band.

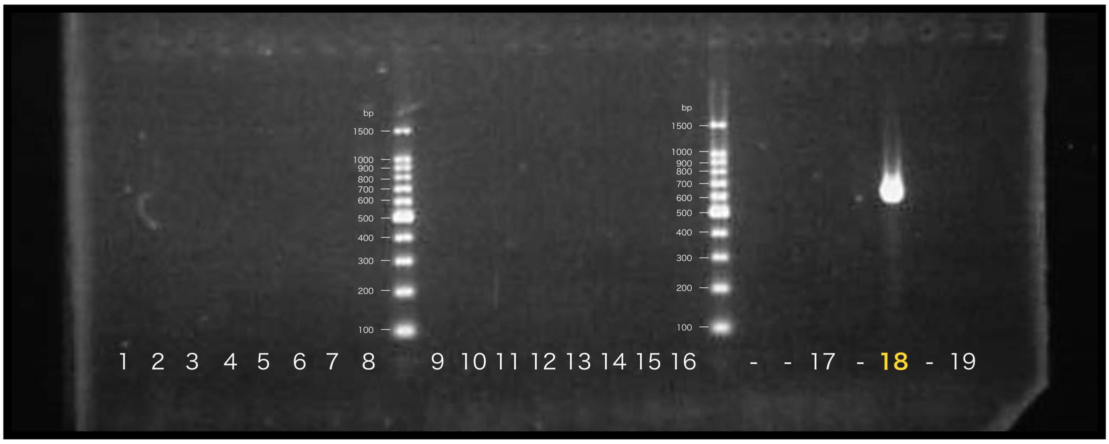

(u) Agarose gel showing results of PCR amplification using primers using Tpi1_F1 and Tpi1_R1 showing the *T. piercei* (lane 19) band.

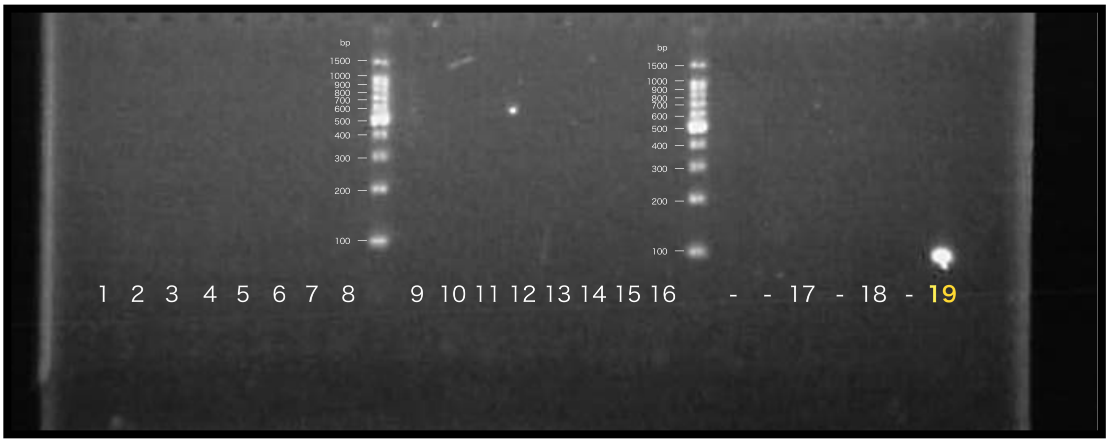

Supplement: S3 File — Lanes 1: P. citri, 2: P. mori, 3: P. ulmi, 4: P. osmanthi, 5: S. shii, 6: E. nomurai, 7: E. celtis, 8: O. castaneae, 9: O. ilicis, 10: O. coffeae, 11: O. gotohi, 12: O. amiensis, 13: T. kanzawai, 14: T. parakanzawai, 15: T. urticae (red form), 16: T. urticae (green form), 17: T. truncatus, 18: T. pueraricola, 19: T. piercei. (PDF) [file pone.0321199.s005.pdf]
