# Supplementary material for: A PCR primer design method for identifying spider mite species using k-mer counting
Source: PLoS One. 2025 Jun 9;20(6):e0321199. doi: 10.1371/journal.pone.0321199 (PMC12148119; doi:10.1371/journal.pone.0321199)

Top: Original gel image of Fig. 3a and S3 File (a), captured using agarose gel electrophoresis and UV transillumination. PCR products were visualized by electrophoresis on an agarose gel, and a 100-bp DNA ladder (Takara Bio, Shiga, Japan) was used as a molecular size marker.

Bottom: Original gel image of S3 File (b), captured using the same method.

Lanes 1: *P. citri*, 2: *P. mori*, 3: *P. ulmi*, 4: *P. osmanthi*, 5: *S. shii*, 6: *E. nomurai*, 7: *E. celtis*, 8: *O. castaneae*, 9: *O. ilicis*, 10: *O. coffeae*, 11: *O. gotohi*, 12: *O. amiensis*, 13: *T. kanzawai*, 14: *T. parakanzawai*, 15: *T. urticae* (red-form), 16: *T. urticae* (green-form) , 17: *T. truncatus* , 18: *T. pueraricola*, 19: *T. piercei*.

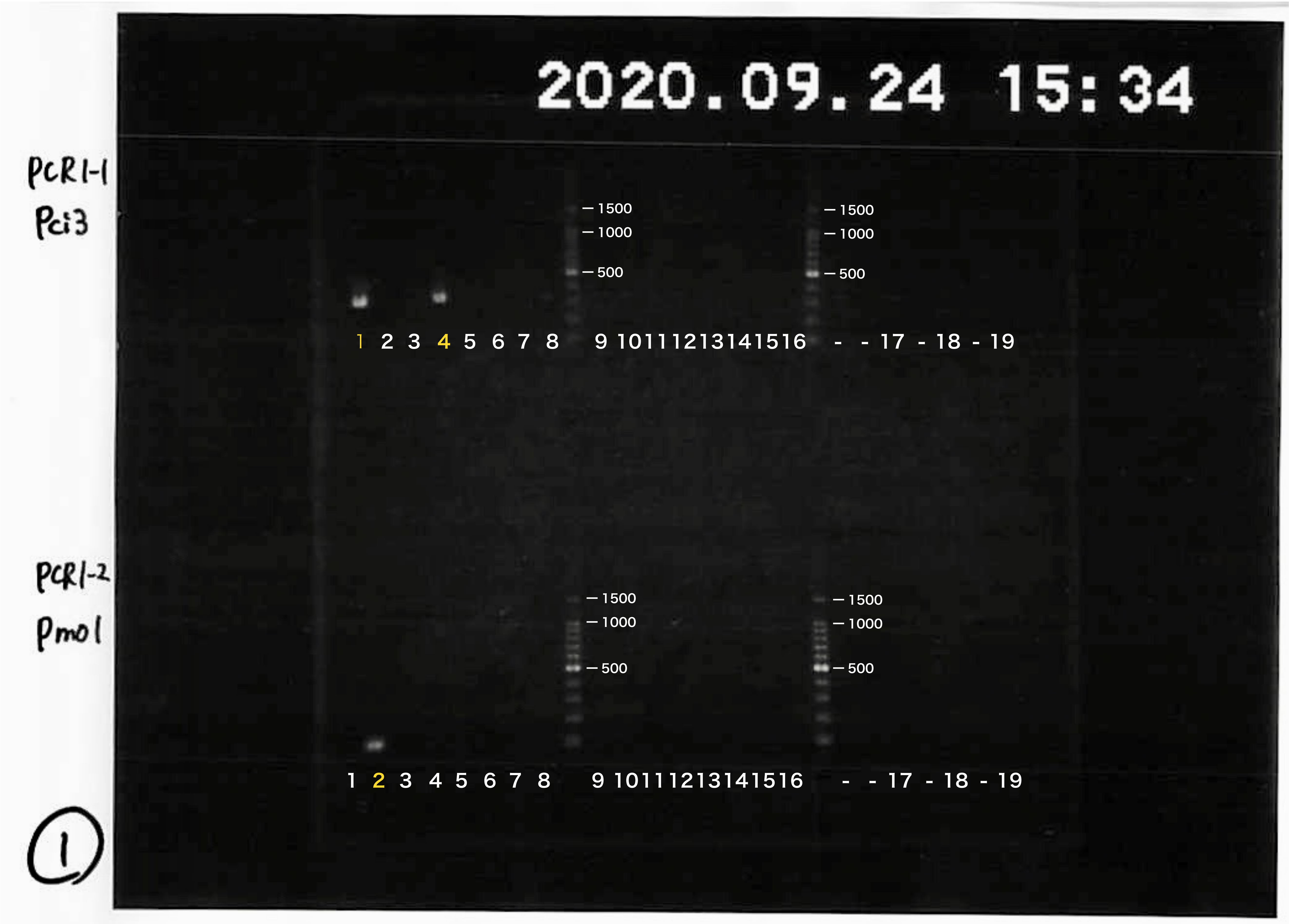

Top: Original gel image of S3 File (c), captured using agarose gel electrophoresis and UV transillumination. PCR products were visualized by electrophoresis on an agarose gel, and a 100-bp DNA ladder (Takara Bio, Shiga, Japan) was used as a molecular size marker.

Bottom: Original gel image of Fig. 3b and S3 File (d), captured using the same method.

Lanes 1: *P. citri*, 2: *P. mori*, 3: *P. ulmi*, 4: *P. osmanthi*, 5: *S. shii*, 6: *E. nomurai*, 7: *E. celtis*, 8: *O. castaneae*, 9: *O. ilicis*, 10: *O. coffeae*, 11: *O. gotohi*, 12: *O. amiensis*, 13: *T. kanzawai*, 14: *T. parakanzawai*, 15: *T. urticae* (red-form), 16: *T. urticae* (green-form) , 17: *T. truncatus* , 18: *T. pueraricola*, 19: *T. piercei*.

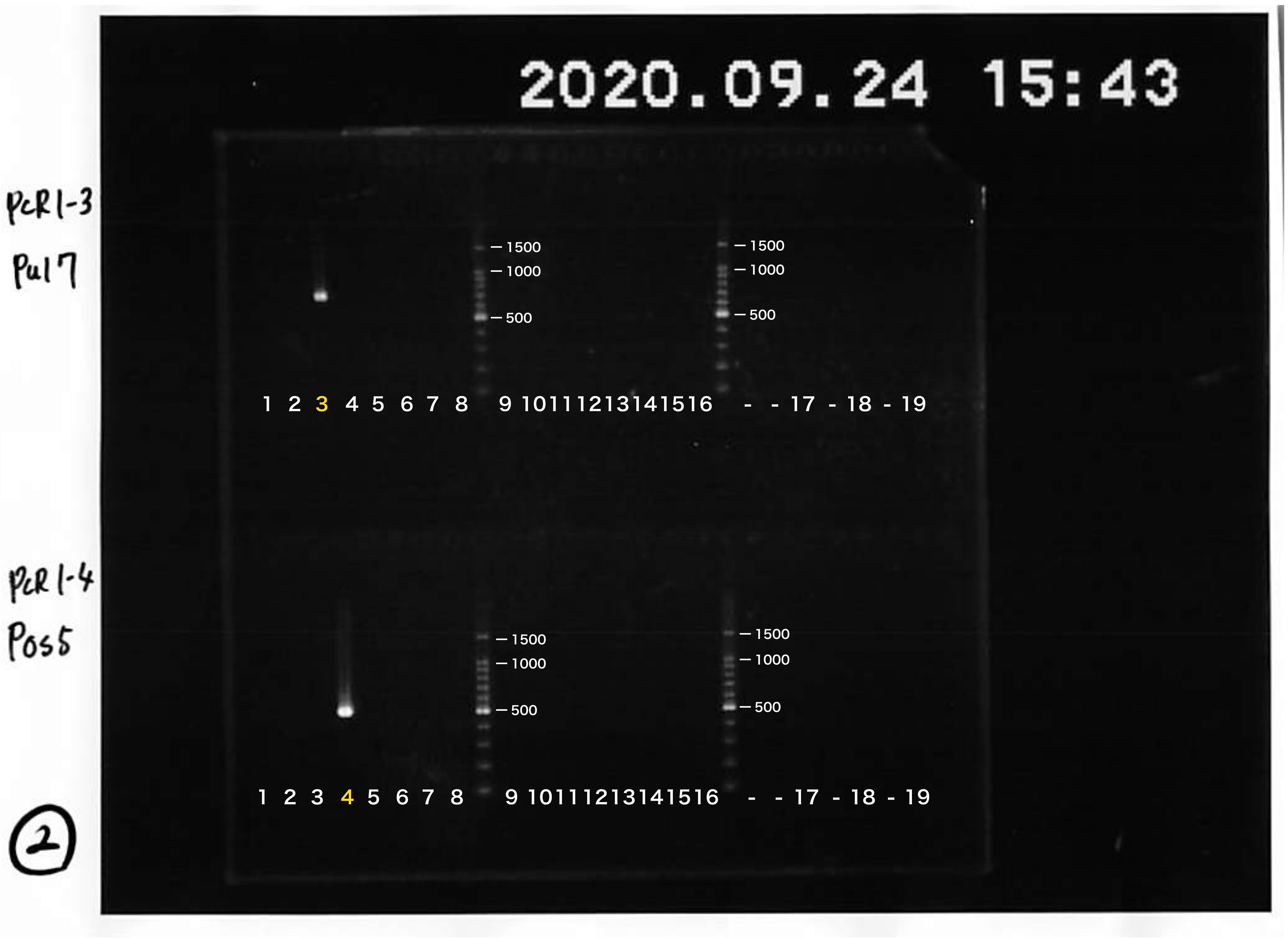

Top: Original gel image of S3 File (e), captured using agarose gel electrophoresis and UV transillumination. PCR products were visualized by electrophoresis on an agarose gel, and a 100-bp DNA ladder (Takara Bio, Shiga, Japan) was used as a molecular size marker.

Bottom: Original gel image of S3 File (f), captured using the same method.

Lanes 1: *P. citri*, 2: *P. mori*, 3: *P. ulmi*, 4: *P. osmanthi*, 5: *S. shii*, 6: *E. nomurai*, 7: *E. celtis*, 8: *O. castaneae*, 9: *O. ilicis*, 10: *O. coffeae*, 11: *O. gotohi*, 12: *O. amiensis*, 13: *T. kanzawai*, 14: *T. parakanzawai*, 15: *T. urticae* (red-form), 16: *T. urticae* (green-form) , 17: *T. truncatus* , 18: *T. pueraricola*, 19: *T. piercei*.

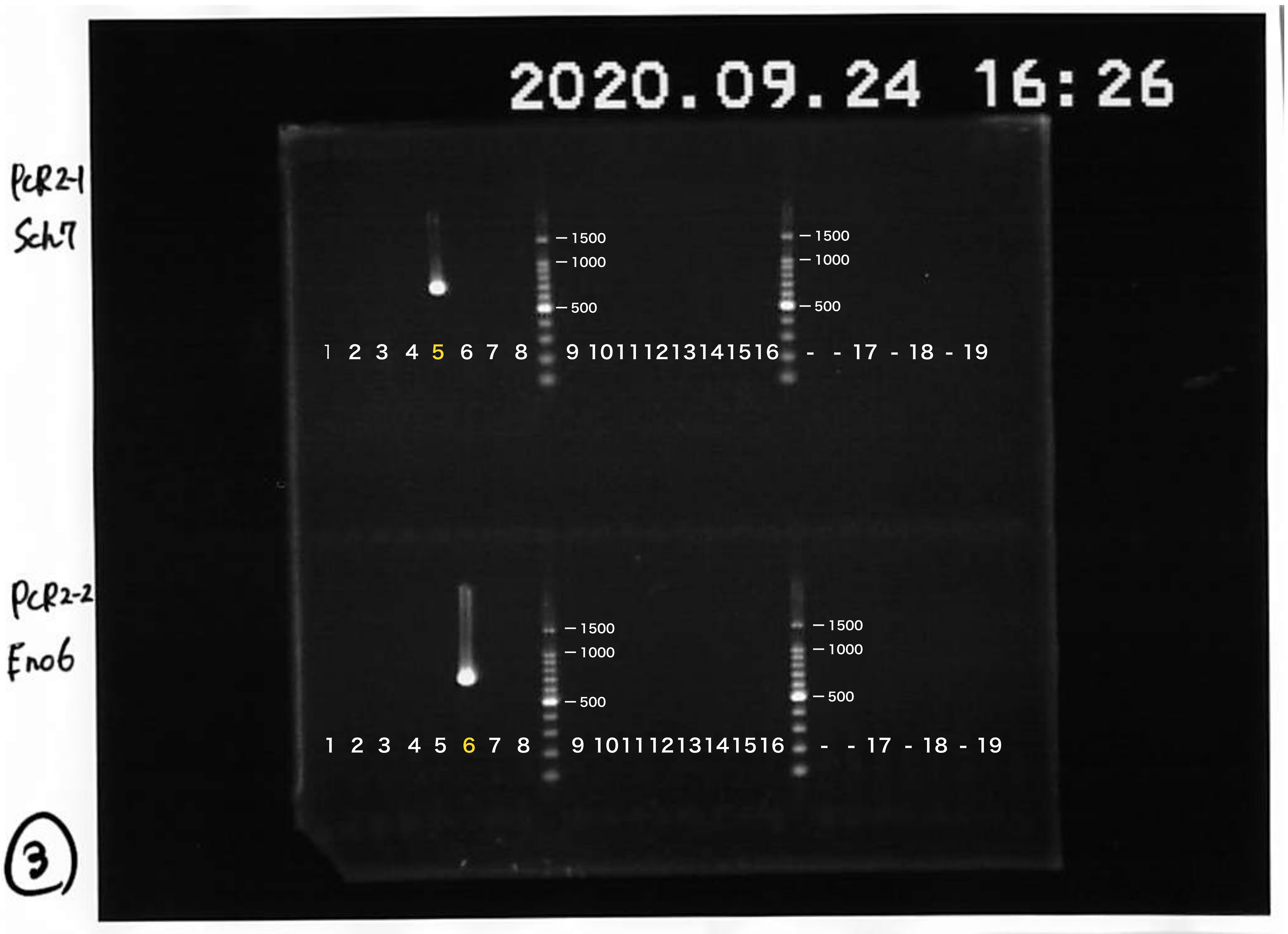

Top: Original gel image of Fig. 3c and S3 File (g), captured using agarose gel electrophoresis and UV transillumination. PCR products were visualized by electrophoresis on an agarose gel, and a 100-bp DNA ladder (Takara Bio, Shiga, Japan) was used as a molecular size marker.

Bottom: Original gel image of S3 File (h), captured using the same method.

Lanes 1: *P. citri*, 2: *P. mori*, 3: *P. ulmi*, 4: *P. osmanthi*, 5: *S. shii*, 6: *E. nomurai*, 7: *E. celtis*, 8: *O. castaneae*, 9: *O. ilicis*, 10: *O. coffeae*, 11: *O. gotohi*, 12: *O. amiensis*, 13: *T. kanzawai*, 14: *T. parakanzawai*, 15: *T. urticae* (red-form), 16: *T. urticae* (green-form) , 17: *T. truncatus* , 18: *T. pueraricola*, 19: *T. piercei*.

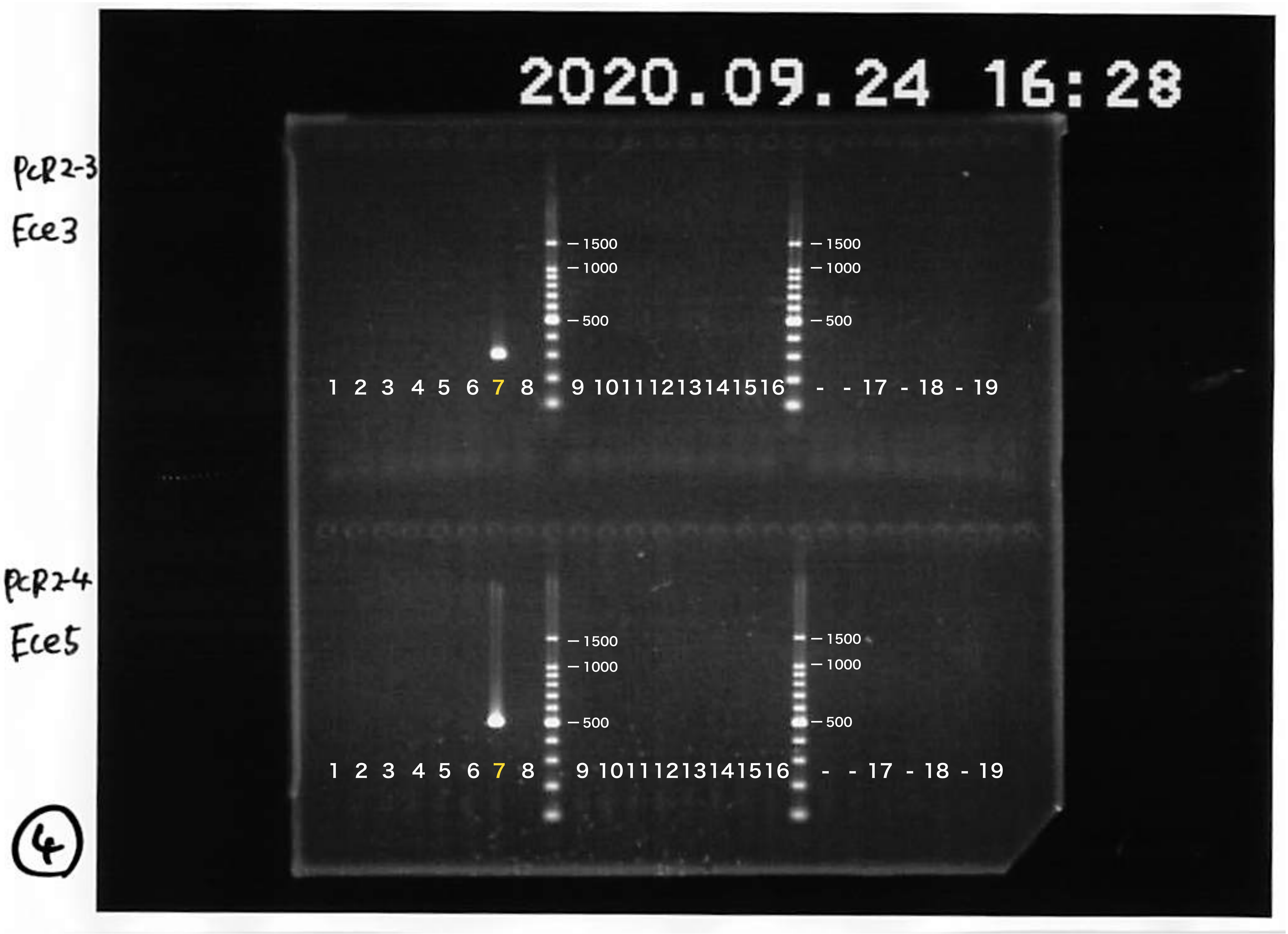

Top: Original gel image of S3 File (i), captured using agarose gel electrophoresis and UV transillumination. PCR products were visualized by electrophoresis on an agarose gel, and a 100-bp DNA ladder (Takara Bio, Shiga, Japan) was used as a molecular size marker.

Bottom: Original gel image of Fig. 3d and S3 File (j), captured using the same method.

Lanes 1: *P. citri*, 2: *P. mori*, 3: *P. ulmi*, 4: *P. osmanthi*, 5: *S. shii*, 6: *E. nomurai*, 7: *E. celtis*, 8: *O. castaneae*, 9: *O. ilicis*, 10: *O. coffeae*, 11: *O. gotohi*, 12: *O. amiensis*, 13: *T. kanzawai*, 14: *T. parakanzawai*, 15: *T. urticae* (red-form), 16: *T. urticae* (green-form) , 17: *T. truncatus* , 18: *T. pueraricola*, 19: *T. piercei*.

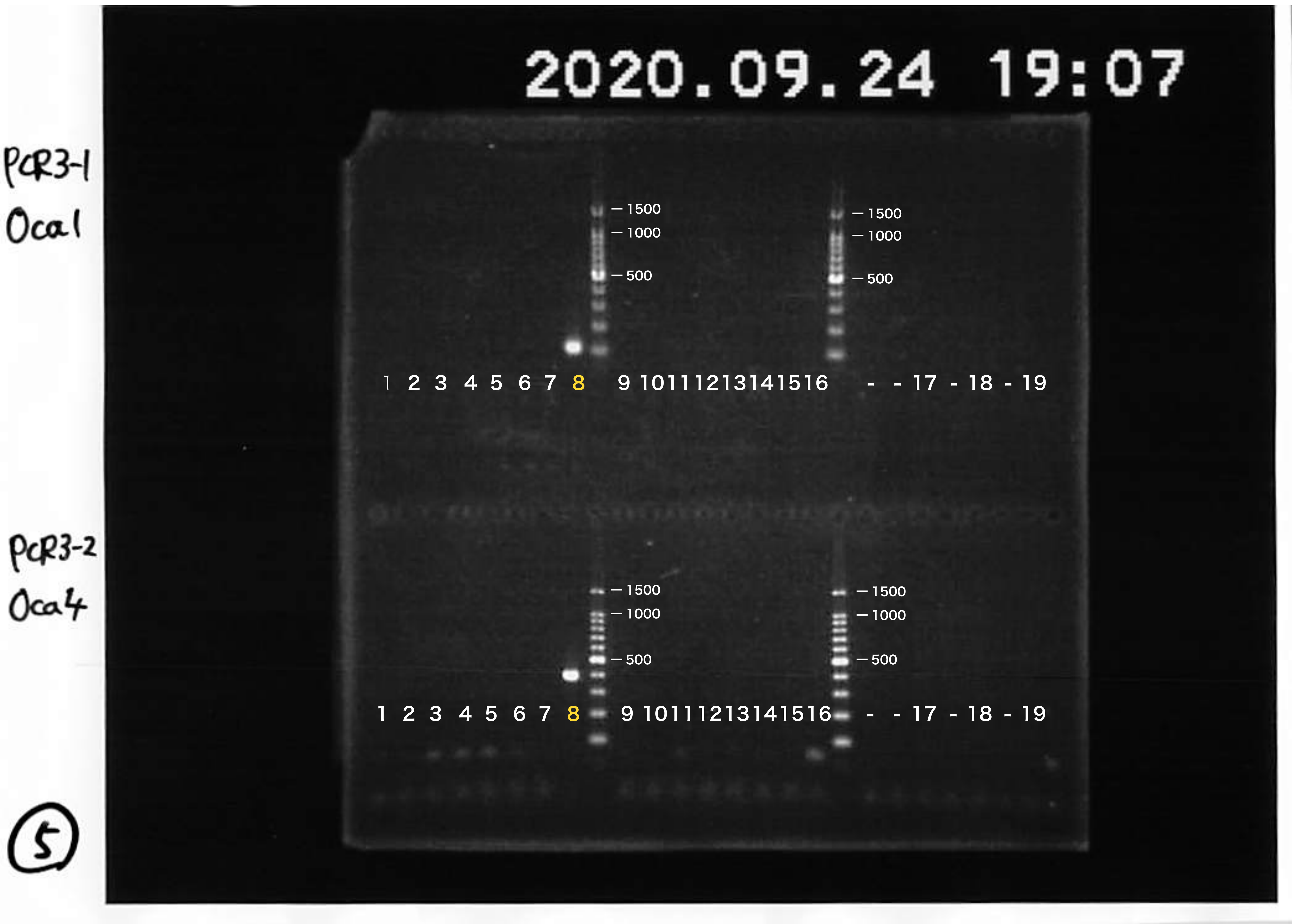

Top: Original gel image of S3 File (k), captured using agarose gel electrophoresis and UV transillumination. PCR products were visualized by electrophoresis on an agarose gel, and a 100-bp DNA ladder (Takara Bio, Shiga, Japan) was used as a molecular size marker.

Bottom: Original gel image of S3 File (l), captured using the same method.

Lanes 1: *P. citri*, 2: *P. mori*, 3: *P. ulmi*, 4: *P. osmanthi*, 5: *S. shii*, 6: *E. nomurai*, 7: *E. celtis*, 8: *O. castaneae*, 9: *O. ilicis*, 10: *O. coffeae*, 11: *O. gotohi*, 12: *O. amiensis*, 13: *T. kanzawai*, 14: *T. parakanzawai*, 15: *T. urticae* (red-form), 16: *T. urticae* (green-form) , 17: *T. truncatus* , 18: *T. pueraricola*, 19: *T. piercei*.

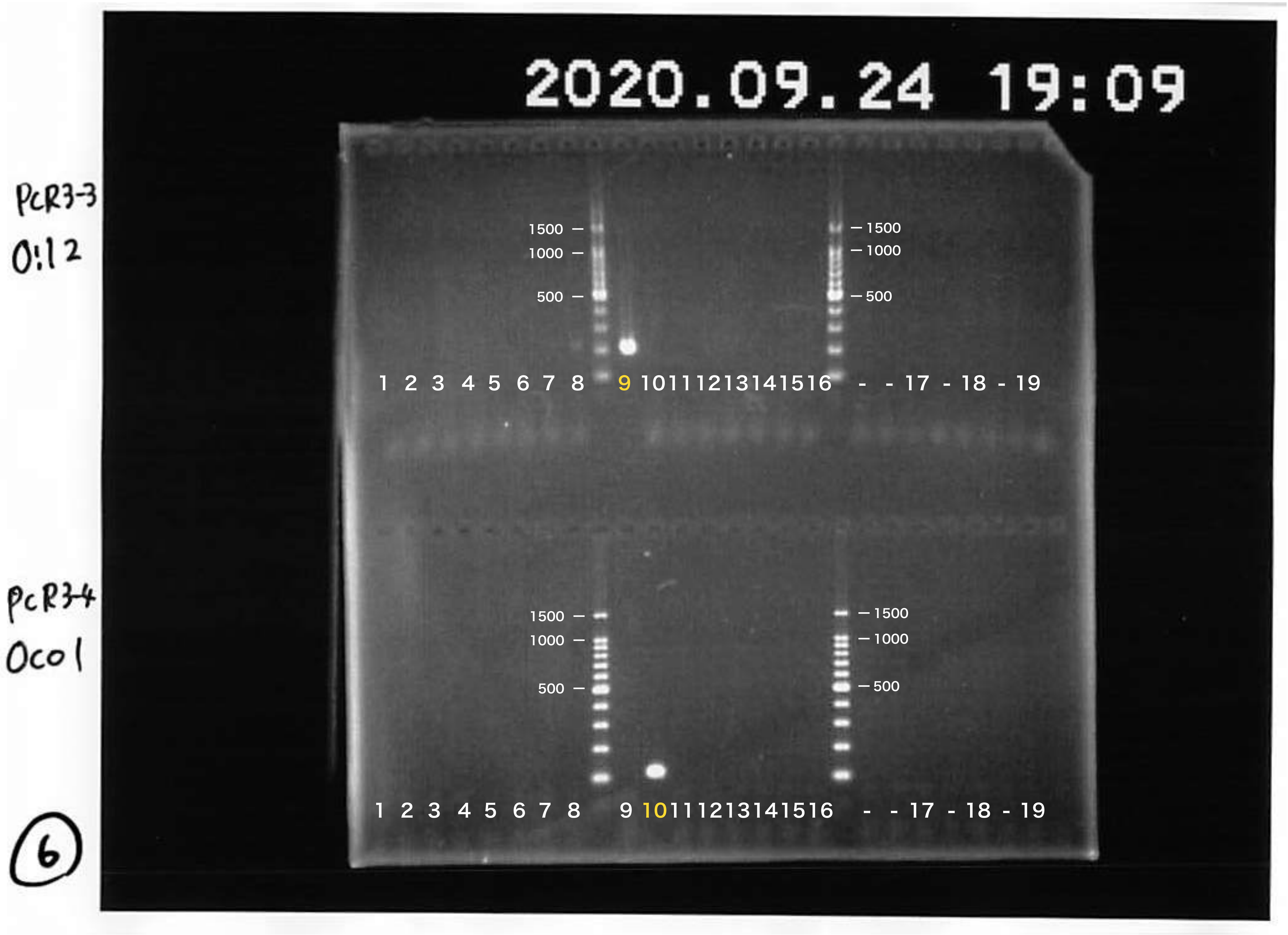

Top: Original gel image of S3 File (m), captured using agarose gel electrophoresis and UV transillumination. PCR products were visualized by electrophoresis on an agarose gel, and a 100-bp DNA ladder (Takara Bio, Shiga, Japan) was used as a molecular size marker.

Bottom: Original gel image of S3 File (n), captured using the same method.

Lanes 1: *P. citri*, 2: *P. mori*, 3: *P. ulmi*, 4: *P. osmanthi*, 5: *S. shii*, 6: *E. nomurai*, 7: *E. celtis*, 8: *O. castaneae*, 9: *O. ilicis*, 10: *O. coffeae*, 11: *O. gotohi*, 12: *O. amiensis*, 13: *T. kanzawai*, 14: *T. parakanzawai*, 15: *T. urticae* (red-form), 16: *T. urticae* (green-form) , 17: *T. truncatus* , 18: *T. pueraricola*, 19: *T. piercei*.

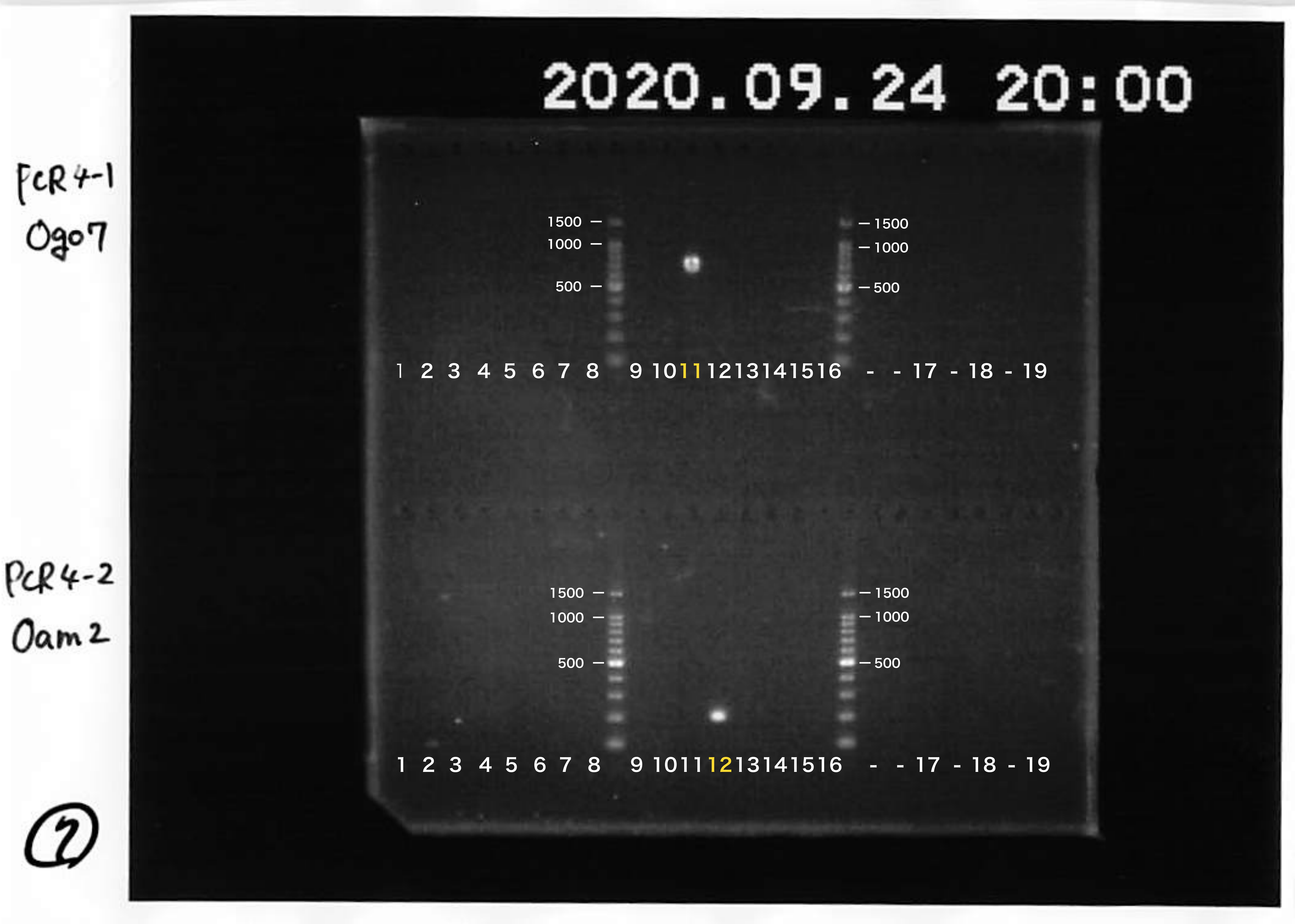

Top: Original gel image of S3 File (o), captured using agarose gel electrophoresis and UV transillumination. PCR products were visualized by electrophoresis on an agarose gel, and a 100-bp DNA ladder (Takara Bio, Shiga, Japan) was used as a molecular size marker.

Bottom: Original gel image of S3 File (p), captured using the same method.

Lanes 1: *P. citri*, 2: *P. mori*, 3: *P. ulmi*, 4: *P. osmanthi*, 5: *S. shii*, 6: *E. nomurai*, 7: *E. celtis*, 8: *O. castaneae*, 9: *O. ilicis*, 10: *O. coffeae*, 11: *O. gotohi*, 12: *O. amiensis*, 13: *T. kanzawai*, 14: *T. parakanzawai*, 15: *T. urticae* (red-form), 16: *T. urticae* (green-form) , 17: *T. truncatus* , 18: *T. pueraricola*, 19: *T. piercei*.

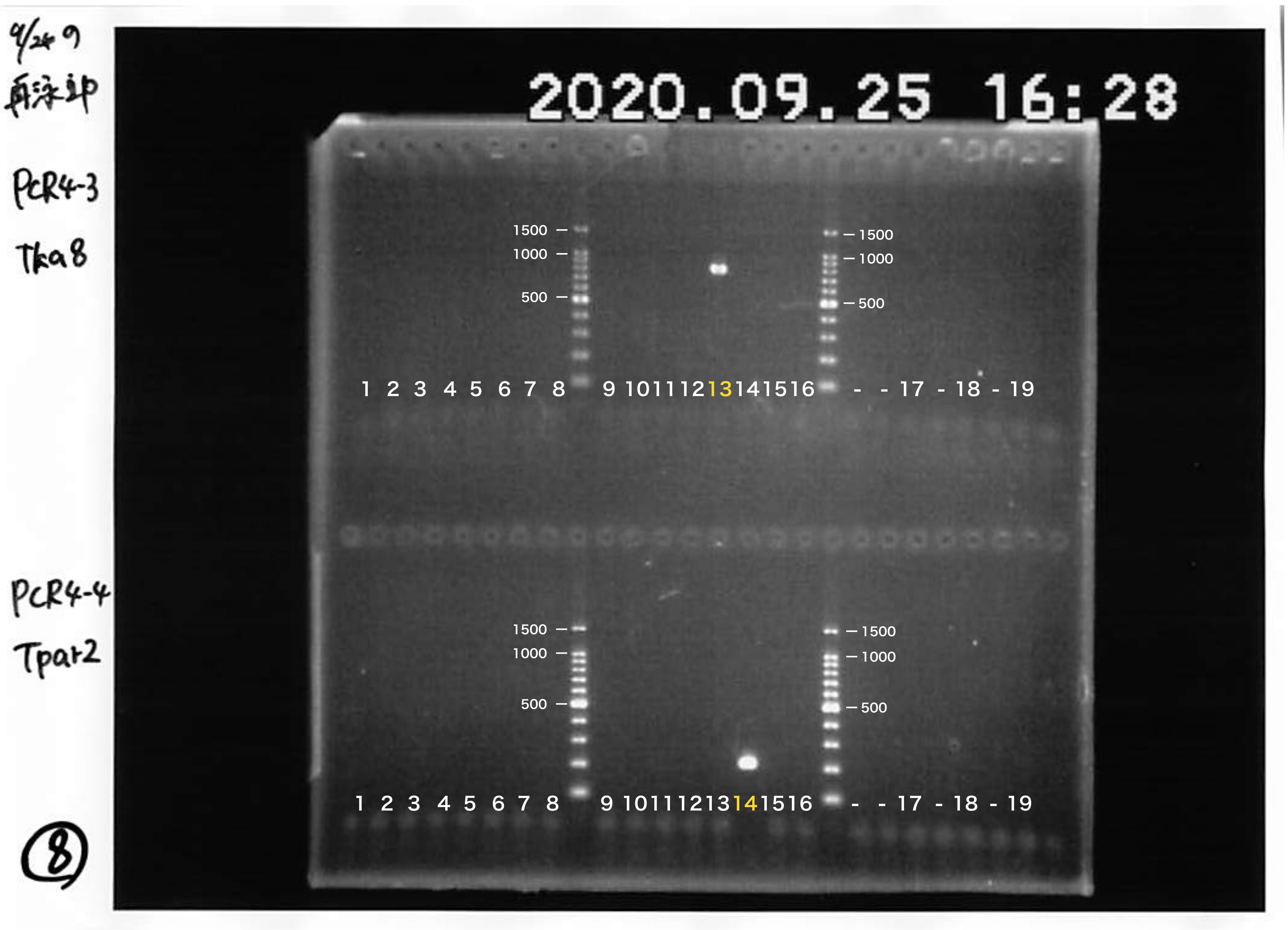

Top: Original gel image of Fig. 3e and S3 File (q), captured using agarose gel electrophoresis and UV transillumination. PCR products were visualized by electrophoresis on an agarose gel, and a 100-bp DNA ladder (Takara Bio, Shiga, Japan) was used as a molecular size marker.

Bottom: This band was excluded from the results because it originated from a sample with ambiguous identification.

Lanes 1: *P. citri*, 2: *P. mori*, 3: *P. ulmi*, 4: *P. osmanthi*, 5: *S. shii*, 6: *E. nomurai*, 7: *E. celtis*, 8: *O. castaneae*, 9: *O. ilicis*, 10: *O. coffeae*, 11: *O. gotohi*, 12: *O. amiensis*, 13: *T. kanzawai*, 14: *T. parakanzawai*, 15: *T. urticae* (red-form), 16: *T. urticae* (green-form) , 17: *T. truncatus* , 18: *T. pueraricola*, 19: *T. piercei*.

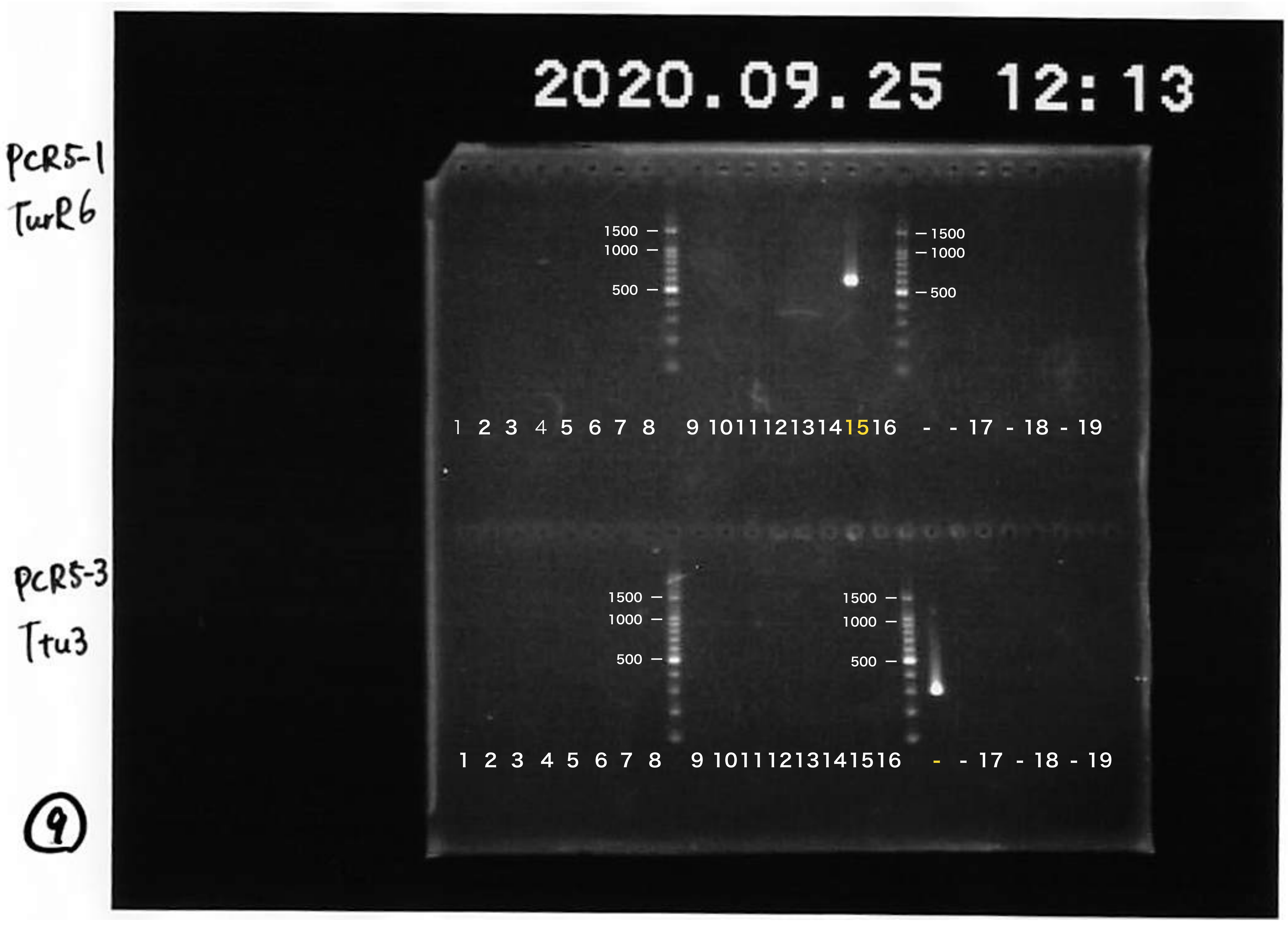

Top: Original gel image of Fig. 3f and S3 File (r), captured using agarose gel electrophoresis and UV transillumination. PCR products were visualized by electrophoresis on an agarose gel, and a 100-bp DNA ladder (Takara Bio, Shiga, Japan) was used as a molecular size marker.

Bottom: This band was excluded from the results because it originated from a sample with ambiguous identification.

Lanes 1: *P. citri*, 2: *P. mori*, 3: *P. ulmi*, 4: *P. osmanthi*, 5: *S. shii*, 6: *E. nomurai*, 7: *E. celtis*, 8: *O. castaneae*, 9: *O. ilicis*, 10: *O. coffeae*, 11: *O. gotohi*, 12: *O. amiensis*, 13: *T. kanzawai*, 14: *T. parakanzawai*, 15: *T. urticae* (red-form), 16: *T. urticae* (green-form) , 17: *T. truncatus* , 18: *T. pueraricola*, 19: *T. piercei*.

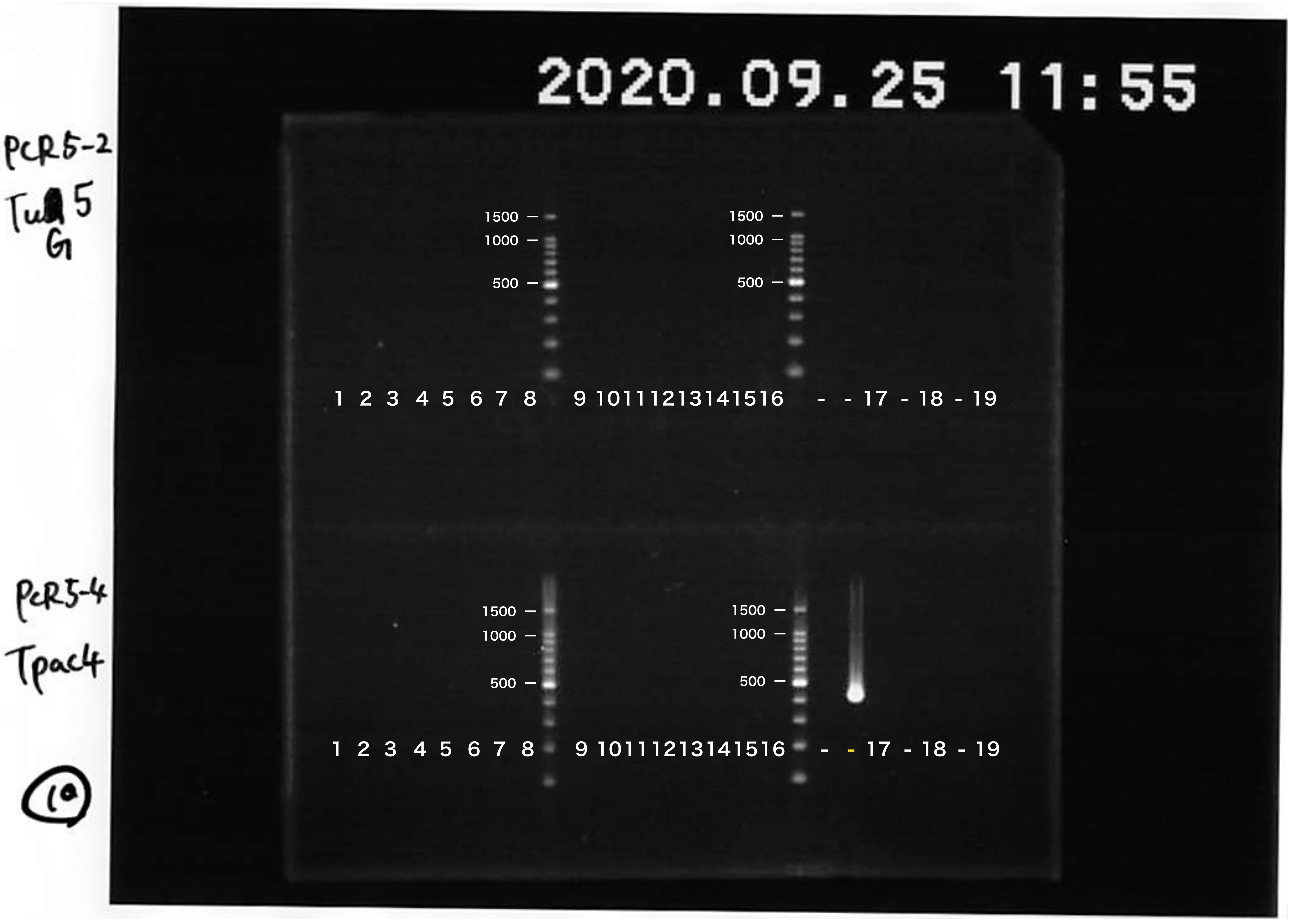

Top: Original gel image of S3 File (s), captured using agarose gel electrophoresis and UV transillumination. PCR products were visualized by electrophoresis on an agarose gel, and a 100-bp DNA ladder (Takara Bio, Shiga, Japan) was used as a molecular size marker.

Bottom: This band was excluded from the results because it originated from a sample with ambiguous identification.

Lanes 1: *P. citri*, 2: *P. mori*, 3: *P. ulmi*, 4: *P. osmanthi*, 5: *S. shii*, 6: *E. nomurai*, 7: *E. celtis*, 8: *O. castaneae*, 9: *O. ilicis*, 10: *O. coffeae*, 11: *O. gotohi*, 12: *O. amiensis*, 13: *T. kanzawai*, 14: *T. parakanzawai*, 15: *T. urticae* (red-form), 16: *T. urticae* (green-form) , 17: *T. truncatus* , 18: *T. pueraricola*, 19: *T. piercei*.

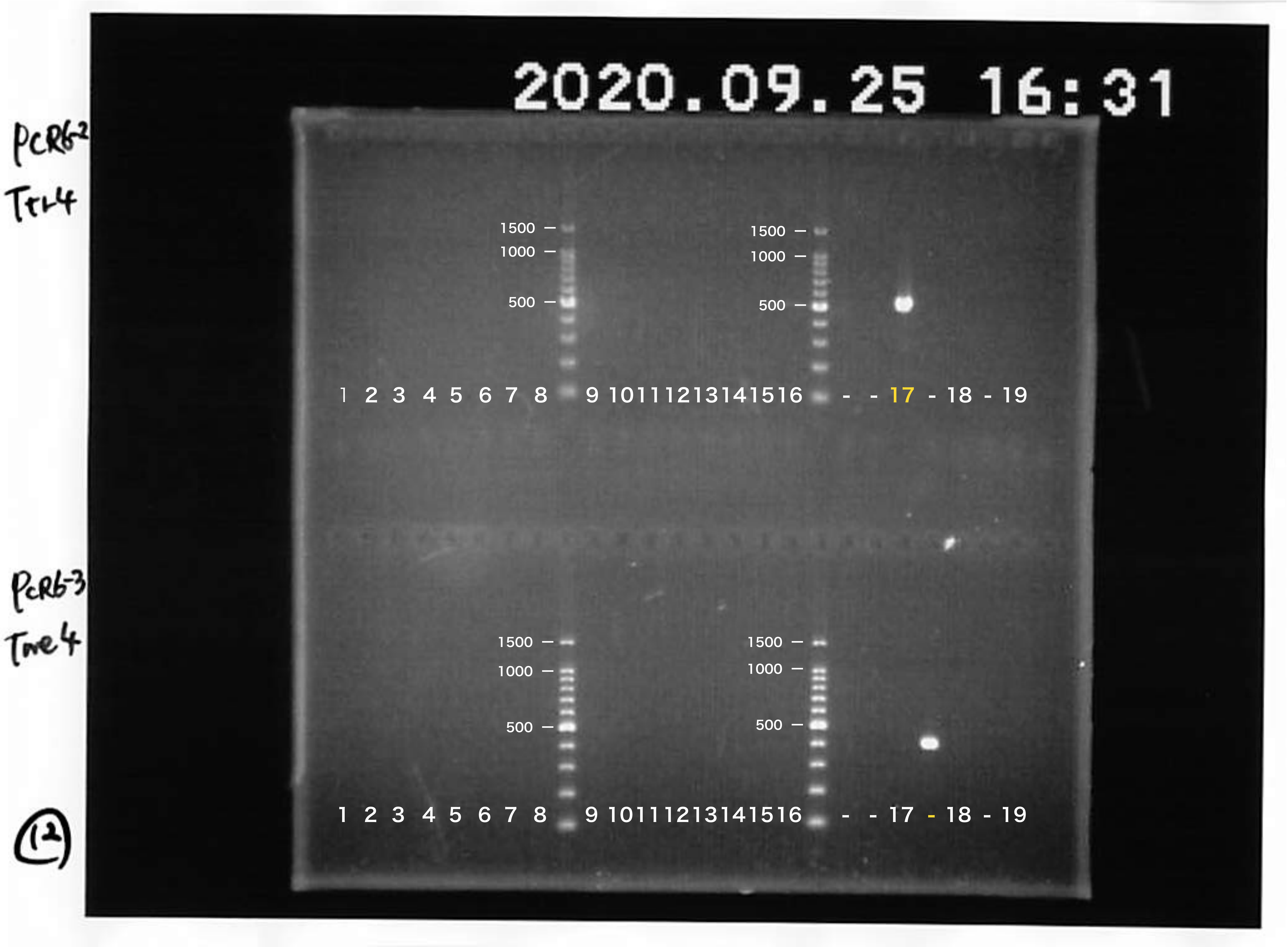

Top: This band was excluded from the results because it originated from a sample with ambiguous identification.

Bottom: Original gel image of S3 File (t), captured using agarose gel electrophoresis and UV transillumination. PCR products were visualized by electrophoresis on an agarose gel, and a 100-bp DNA ladder (Takara Bio, Shiga, Japan) was used as a molecular size marker.

Lanes 1: *P. citri*, 2: *P. mori*, 3: *P. ulmi*, 4: *P. osmanthi*, 5: *S. shii*, 6: *E. nomurai*, 7: *E. celtis*, 8: *O. castaneae*, 9: *O. ilicis*, 10: *O. coffeae*, 11: *O. gotohi*, 12: *O. amiensis*, 13: *T. kanzawai*, 14: *T. parakanzawai*, 15: *T. urticae* (red-form), 16: *T. urticae* (green-form) , 17: *T. truncatus* , 18: *T. pueraricola*, 19: *T. piercei*.

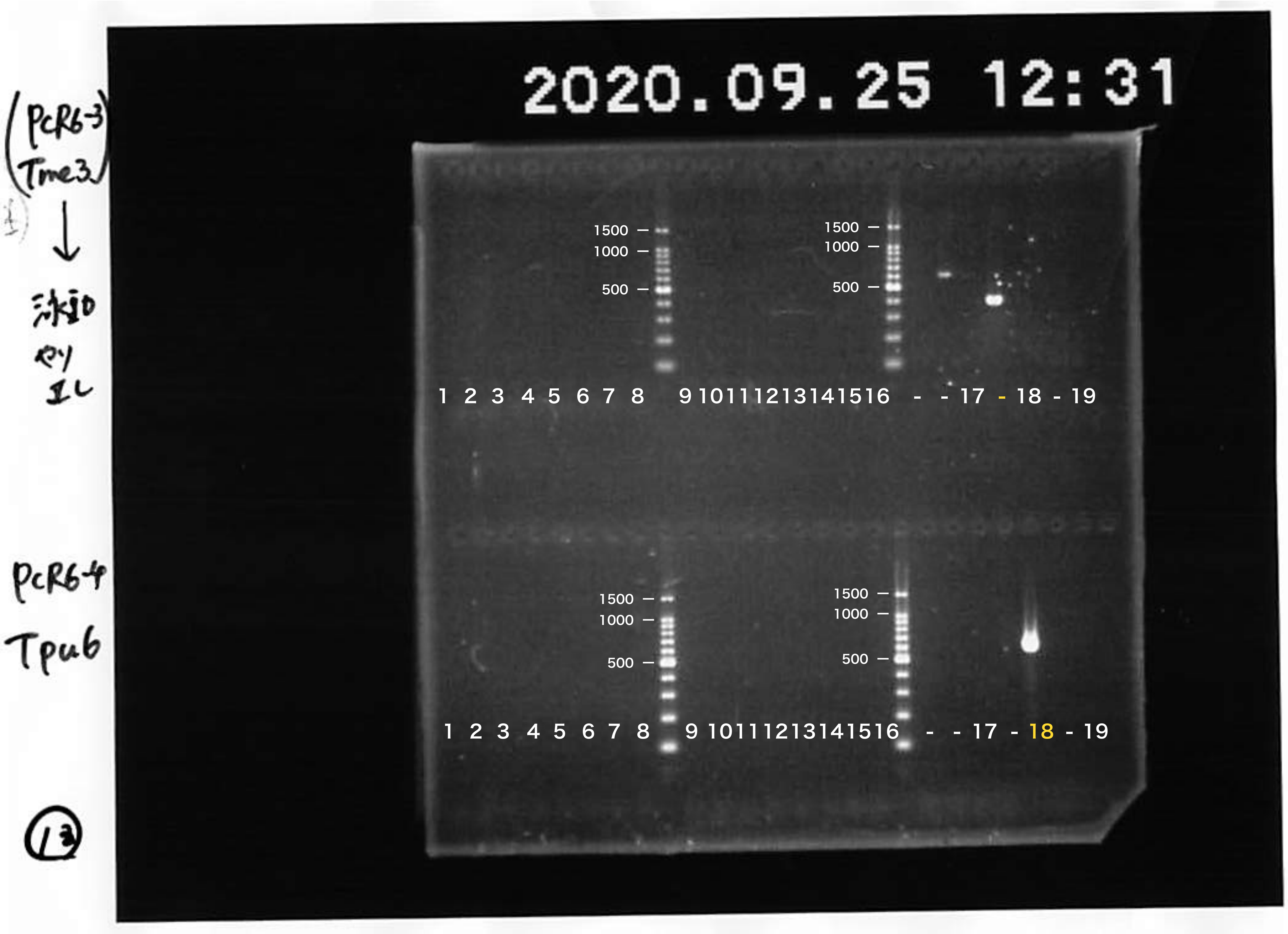

Top: This band was excluded from the results because it originated from a sample with ambiguous identification.

Bottom: Original gel image of S3 File (u), captured using agarose gel electrophoresis and UV transillumination. PCR products were visualized by electrophoresis on an agarose gel, and a 100-bp DNA ladder (Takara Bio, Shiga, Japan) was used as a molecular size marker.

Lanes 1: *P. citri*, 2: *P. mori*, 3: *P. ulmi*, 4: *P. osmanthi*, 5: *S. shii*, 6: *E. nomurai*, 7: *E. celtis*, 8: *O. castaneae*, 9: *O. ilicis*, 10: *O. coffeae*, 11: *O. gotohi*, 12: *O. amiensis*, 13: *T. kanzawai*, 14: *T. parakanzawai*, 15: *T. urticae* (red-form), 16: *T. urticae* (green-form) , 17: *T. truncatus* , 18: *T. pueraricola*, 19: *T. piercei*.

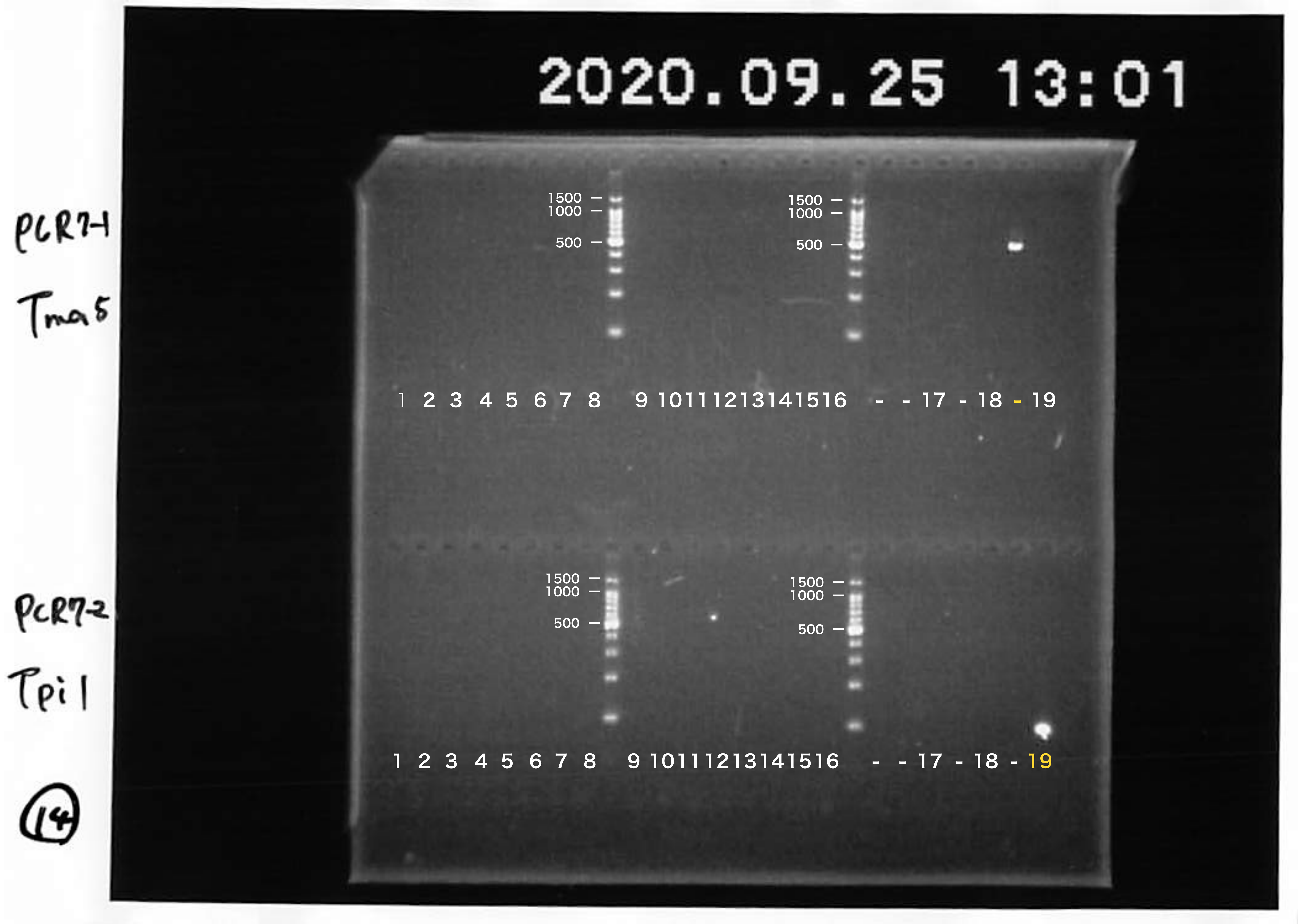

Supplement: S1 Raw Images — (PDF) [file pone.0321199.s006.pdf]
