# Supplementary material for: A PCR primer design method for identifying spider mite species using k-mer counting
Source: PLoS One. 2025 Jun 9;20(6):e0321199. doi: 10.1371/journal.pone.0321199 (PMC12148119; doi:10.1371/journal.pone.0321199)
Supplement: Attachment — Submitted filename: Matsuda review.pdf [file pone.0321199.s007.pdf]

The authors present a computational method to identify species-specific PRC primer pairs from RNA-Seq data to distinguish between different species of spider mites. They then experimentally test the primer pairs on each of 19 species to ensure that A) there is amplification in the target species and B) there is no amplification in any of the non-target species. I believe the authors achieve their aims and I have to reason to doubt the reproducibility of their results. However, in places the manuscript would benefit from more motivation of the approach the authors took and consideration or discussion of alternatives. Moreover, I think the computational pipeline could be simplified.

I do not have sufficient expertise to judge the lab protocols and will focus this review on the computational methods and the study set-up in general.

1. Consider changing the title to "A PCR primer design method for identifying spider mite species using *k*-mers" or "... using *k*-mer counts" or "... using *k*-mer counting". There are several algorithms for *k*-mer counting and there is no discussion of those in the paper and that is also not necessary. What matters for the paper is the *concept* of unique *k*-mers, not the algorithm used to identify those.
2. Describe literature for species diagnostic PCR and the way it is used to diagnose species. There are instances where the PCR products have different known lengths for the different species and the diagnosis is based on comparing the query length to the expected lengths for each species. There are instances where the PCR product is sequenced and certain known variants are used to diagnose the species. In this case, the PCR primers should only work in a single species so whether or not PCR amplification is observed is used to diagnose species.
3. Motivate why the primers are designed from RNA-Seq data and not from whole genome short read data. The authors briefly mention why they don't work with genome assemblies (because these are available for fewer species than the RNA-Seq data), but they do not consider whole genome DNA data (without necessarily making this into a genome assembly, but rather work with the reads directly or aligned to an available genome assembly of a related species). Using whole genome data would probably result in more available targets because A) a lot more sequence is considered and B) non-transcribed regions are often less constrained in accumulating variation so likely there are many more species-informative regions among non-transcribed regions.
4. The methods are a bit difficult to follow. To my understanding, RNA reads are assembled and contigs would represent an observed transcript. Then, by orthology search from the annotated *T. urticae* genome, those contigs corresponding to an intronless gene are selected, and alignments of the 19 species are generated for each gene. On those selected contigs, *30*-mers are counted and unique *30*-mers were used for primer design. The paired primer was selected manually.
    a. Why are alignments done separately for CDS and 5' and 3' sequence, also using different alignment methods?
    b. In figure 2 there seems to be a step missing, the phylogenetic trees are constructed to distinguish orthologous genes from paralogous genes, true? Also please mention what the distinguishing characteristic is between orthologous and paralogous genes in a phylogenetic tree (now it just says 'identified visually').
    c. In the **Extracting unique *k*-mers** section the criteria could be ordered differently for ease of understanding.
        i. Start with requirement 9, which is the crucial step, namely retain only unique *k*-mers (unique within and across species). NB: the 'and' should be replaced by 'or' to achieve (unique within and across species).
        ii. Then introduce/motivate the remaining requirements: they are important for primer binding/open chromatin or other reasons why they were implemented.

iii. In requirement 6,7,8 replace ‘and’ by ‘or’. A number cannot be smaller than 11 and greater than 17 at the same time.
iv. Add ‘primer melting Temperature’ in brackets after Tm.

d. In the **Filtering *k*-mers** section, it seems a bit roundabout to use bowtie and blastn to filter *k*-mers for similarity to the contigs. Under the hood this can probably also be phrased as a *k*-mer matching criterion. I’m curious to know whether you really need bowtie and blast to get a well filtered set or whether by tweaking the parameters you can get the same level of filtering by just using one of these programs.

5. It is unclear how the paired primer is designed ‘manually from the alignments’. Please elaborate on what selection criteria were used (upstream/downstream from candidate; distance from candidate; similarity/differentiation between species; uniqueness in transcriptome; primer length, etc.). Why were not simply pairs of species-specific *k*-mers occurring on the same contig selected (with potentially additional selection criteria)?
6. Potentially related to the point above: it is unclear how the primer pairs for experimental testing were selected from the tens, hundreds or thousands of candidate targets per species. Were all candidates for which a paired target could be identified selected? Or only one or two per species? If the latter, how were those selected?
7. What would be the effect of within-species variation? Here, 100-200 individuals are pooled for RNA extraction. How would the contig assembly and *k*-mer counts be affected by genetic variants within these individuals? (Also, related to point 3, this would probably be a benefit of using RNA data over DNA data, exactly because transcribed regions are more constrained in accumulating variation). Please discuss.
8. With about 1200 species in the Tetranychidae family there are many species not represented among the set of primers designed in this study. What do we expect to happen if applying this method to species not included in the design? Will none of the primer pairs amplify? Or only one pair of the closest related species? And if so, would the product have the same length or different? And then, would there be any way to tell this unrepresented species from the target species? Or would multiple primer pairs amplify? What are the implications for interpreting the data?
9. Is there a way to build in a positive control for this design? It seems relevant that when the diagnosis is based on the absence of a PCR band (diagnosis being ‘this is not the target species’) there is a way to check that the PCR actually worked.
10. Would the authors offer a hypothesis as to why some primer pairs fail to amplify anything at all?
11. As the authors mention, multiplex PCR would make this species diagnosis method a lot less labour-intensive. Maybe this is a naive question (as I don’t have a lot of wet-lab expertise) but would the authors give multiplexing a try? Or if not, could they mention in the discussion which difficulties or challenges they foresee in adapting the protocol to multiplex PCR?
