# Supplementary material for: A PCR primer design method for identifying spider mite species using k-mer counting
Source: PLoS One. 2025 Jun 9;20(6):e0321199. doi: 10.1371/journal.pone.0321199 (PMC12148119; doi:10.1371/journal.pone.0321199)
Supplement: Attachment — Submitted filename: Response to Reviewers.docx [file pone.0321199.s009.docx]

Dear Reviewers,

We sincerely thank you for your thorough review and constructive comments on our manuscript. Your valuable suggestions have greatly contributed to improving the quality and clarity of our work.

In this document, we have provided detailed responses to each of your comments. We have carefully addressed all points and revised the manuscript accordingly. Below, we outline our responses to your comments, with changes in the manuscript highlighted in the revised version.

Sincerely,

Tomoko Matsuda

Nihon BioData Corporation

-------------------------------------------------------------------------------------------------------------------------------------------

Comments 1

1. Consider changing the title to “A PCR primer design method for identifying spider mite species using *k*-mers” or “... using *k*-mer counts” or “... using *k*-mer counting”. There are several algorithms for *k*-mer counting and there is no discussion of those in the paper and that is also not necessary. What matters for the paper is the concept of unique *k*-mers, not the algorithm used to identify those.

Response 1

We revised the title accordingly. Please refer to line 4.

Before Revision

A PCR primer design method for identifying spider mite species using the *k*-mer counting algorithm

After Revision (line 4, Revised MS (clean copy))

A PCR primer design method for identifying spider mite species using the *k*-mer counting

-------------------------------------------------------------------------------------------------------------------------------------------

Comments 2

2. Describe literature for species diagnostic PCR and the way it is used to diagnose species. There are instances where the PCR products have different known lengths for the different species and the diagnosis is based on comparing the query length to the expected lengths for each species. There are instances where the PCR product is sequenced and certain known variants are used to diagnose the species. In this case, the PCR primers should only work in a single species so whether or not PCR amplification is observed is used to diagnose species.

Response 2

The points you raised regarding the use of species- specific diagnostic PCR methods have been incorporated into the manuscript. Please refer to line 37.

Before Revision

Among its many uses, the polymerase chain reaction (PCR) can be used for distinguishing morphologically similar species. Designing the PCR primers to be specific for the target is essential.

After Revision (line 37, Revised MS (clean copy)))

Polymerase chain reaction (PCR) is a method for amplifying DNA fragments *in* *vitro* [1]. Because of its convenience and low cost, PCR has become a standard technique not only for biomedical, infectious disease, and forensic applications but also in other fields of biology, such as agricultural and ecological research. Among its many uses, PCR can be used to distinguish morphologically similar species. For example, PCR-RFLP (restriction fragment length polymorphism) distinguishes closely related species by using a single restriction enzyme to produce fragments of different lengths from DNA markers [2-4]. Another method is to design species-specific primers that can only be amplified in certain species and to identify the species based on successful PCR amplification and correct product size [5-7].

1. Mullis K, Faloona F. Specific synthesis of DNA in vitro via a polymerase-catalyzed chain reaction. Methods Enzymol. 1987;155: 335–350.

2. Clark TL, Meinke LJ, Foster JE. PCR–RFLP of the mitochondrial cytochrome oxidase (subunit I) gene provides diagnostic markers for selected *Diabrotica* species (Coleoptera: Chrysomelidae). Bull Entomol Res. 2001;91: 419–427.

3. Asraoui JF, Sayar NP, Knio KM, Smith CA. Fly diversity revealed by PCR-RFLP of mitochondrial DNA. Biochem Mol Biol Educ. 2008;36: 354–362.

4. Chua TH, Chong YV, Lim SH. Species determination of Malaysian *Bactrocera* pests using PCR-RFLP analyses (Diptera: Tephritidae). Pest Manag Sci. 2010;66: 379–384.

5. Lu W-N, Wu Y-T, Kuo M-H. Development of species-specific primers for the identification of aphids in Taiwan. Appl Entomol Zool (Jpn). 2008;43: 91–96.

6. Zhang T, Wang Y-J, Guo W, Luo D, Wu Y, Kučerová Z, et al. DNA barcoding, species-specific PCR and real-time PCR techniques for the identification of six *Tribolium* pests of stored products. Sci Rep. 2016;6: 28494.

7. Zhao Z-H, Cui B-Y, Li Z-H, Jiang F, Yang Q-Q, Kučerová Z, et al. The establishment of species-specific primers for the molecular identification of ten stored-product psocids based on ITS2 rDNA. Sci Rep. 2016;6: 21022.

-------------------------------------------------------------------------------------------------------------------------------------------

Comments 3

3. Motivate why the primers are designed from RNA-Seq data and not from whole genome short read data. The authors briefly mention why they don’t work with genome assemblies (because these are available for fewer species than the RNA-Seq data), but they do not consider whole genome DNA data (without necessarily making this into a genome assembly, but rather work with the reads directly or aligned to an available genome assembly of a related species). Using whole genome data would probably result in more available targets because A) a lot more sequence is considered and B) non-transcribed regions are often less constrained in accumulating variation so likely there are many more species-informative regions among non-transcribed regions.

Response 3

We have added additional information about whole-genome short-read data in the Introduction section. Please refer to line 69.

Before Revision

On the other hand, transcriptome RNA-Seq data for more than 50 species in the family Tetranychidae are publicly available [22].

After Revision (line 69, Revised MS (clean copy))

Using whole genome data would likely increase the number of available targets and the number of sequences and non-transcribed regions considered. However, publicly available sequencing resources for spider mites (Acari, Tetranychidae) are limited, with only 13 species having whole genome short-read data deposited in the SRA, compared to 79 species with available RNA-Seq data [29] (according to the NCBI SRA database accessed on 24 December 2024). In a previous study, we performed RNA-Seq analysis on 72 species (73 strains) of spider mites. The resulting data have been made publicly available in DRA/SRA/ERA databases under the accession number DRA007145.

-------------------------------------------------------------------------------------------------------------------------------------------

Comments 4-a

4. The methods are a bit difficult to follow. To my understanding, RNA reads are assembled and contigs would represent an observed transcript. Then, by orthology search from the annotated *T*. *urticae* genome, those contigs corresponding to an intronless gene are selected, and alignments of the 19 species are generated for each gene. On those selected contigs, 30-mers are counted and unique 30-mers were used for primer design. The paired primer was selected manually.

a. Why are alignments done separately for CDS and 5’ and 3’ sequence, also using different alignment methods?

Response 4-a

It is common practice to align coding sequences (CDSs) and untranslated regions (UTRs) separately, as demonstrated in the following previous studies. This approach is necessary because obtaining full-length transcripts across all species is challenging when using Illumina short-read sequencing technology.

Chua, B. H., McMinn, P. C., Lam, S. K. & Chua, K. B. Comparison of the complete nucleotide sequences of echovirus 7 strain UMMC and the prototype (Wallace) strain demonstrates significant genetic drift over time. J. Gen. Virol. 82, 2629–2639 (2001).

Wang, X.-W. et al. Transcriptome analysis and comparison reveal divergence between two invasive whitefly cryptic species. BMC Genomics 12, 458 (2011).

Cheng, J., Zhang, L., Hui, M., Li, Y. & Sha, Z. Insights into adaptive divergence of Japanese mantis shrimp Oratosquilla oratoria inferred from comparative analysis of full-length transcriptomes. Front. Mar. Sci. 9, 975686 (2022).

CDSs were aligned using DIALIGN-TX, which accounts for amino acid sequences to improve alignment accuracy. In contrast, 3' and 5' UTR were aligned using MAFFT, a widely used alignment tool, as considering amino acid sequences is not necessary for these non-coding regions. We have mentioned to these in the Materials & Methods section. Please refer to line 201.

Added text (line 201, Revised MS (clean copy))

CDSs were aligned using DIALIGN-TX, which considers amino acid sequences to improve alignment accuracy, as described in the section 'Identification of putative orthologous genes.' In contrast, 3' and 5' UTR sequences were aligned using MAFFT [56], a widely used alignment tool, as considering amino acid sequences is not necessary for these non-coding regions.

-------------------------------------------------------------------------------------------------------------------------------------------

Comments 4-b

b. In figure 2 there seems to be a step missing, the phylogenetic trees are constructed to distinguish orthologous genes from paralogous genes, true? Also please mention what the distinguishing characteristic is between orthologous and paralogous genes in a phylogenetic tree (now it just says ‘identified visually’).

Response 4-b

Despite recent advances, all existing ortholog detection methods still suffer from false positive and false negative rates. For example, OrthoFinder is widely used to estimate orthologous genes, providing robust phylogenetic inference and orthogroup identification. However, by definition, orthogroups identified using this method may contain both orthologs and paralogs, as OrthoFinder groups all related sequences together without fully distinguishing between the two. Therefore, it is not always possible to completely exclude paralogs entirely using this approach.

We therefore constructed a phylogenetic tree to distinguish between orthologous and paralogous genes. A gene was classified as a paralog if its phylogenetic relationship clearly deviated from the established relationships within known spider mite family Tetranychidae

For example, as illustrated in the figure below, if the phylogenetic tree showed that the sequence from "*T*. *truncatus* (1765_T_tru_com938.seq3.p1)" does not cluster with sequences from other species in a manner consistent with their known evolutionary relationships, it was identified as a paralog rather than an ortholog. If a phylogenetic tree included paralogs, the genes used to construct that tree were excluded from the analysis. This visual assessment was based on the topology of the tree and the divergence of specific clades.

We have added these to the Materials & Methods section. Please refer to line 144.

Before Revision

Then, phylogenetic trees based on each orthologous gene were constructed using RAxML [45].

After Revision (line 144, Revised MS (clean copy))

Despite recent advances, existing ortholog detection methods still suffer from false-positive and false-negative rates. We therefore constructed a phylogenetic tree using RAxML [52] to differentiate between orthologous and paralogous genes. A gene was classified as a paralog if its phylogenetic relationship distinctly deviated from the established relationships among known spider mite species in the family Tetranychidae [29].

-------------------------------------------------------------------------------------------------------------------------------------------

Comments 4-c

c. In the Extracting unique *k*-mers section the criteria could be ordered differently for ease of understanding.

i. Start with requirement 9, which is the crucial step, namely retain only unique *k*-mers (unique within and across species). NB: the ‘and’ should be replaced by ‘or’ to achieve (unique within and across species).

ii. Then introduce/motivate the remaining requirements: they are important for primer binding/open chromatin or other reasons why they were implemented.

iii. In requirement 6,7,8 replace ‘and’ by ‘or’. A number cannot be smaller than 11 and greater than 17 at the same time.

iv. Add ‘primer melting Temperature’ in brackets after Tm.

Response 4-c

We agree with your observations. As a result, we have revised the structure of these sections to follow a more logical order, ensuring a clearer presentation of each item and its purpose. Please refer to line 158.

Before Revision

The *k*-mers obtained from Jellyfish were filtered by the following requirements.

1. When a *k*-mer contained four or more runs of a single base (e.g., AAAAA and GGGG), the *k*-mer was removed.

2. When both 5' and 3' ends of a *k*-mer were A or T, the *k*-mer was removed.

3. When both 5' and 3' ends of a *k*-mer were three or more of G or C, the *k*-mer was removed.

4. When both 5' and 3' ends of a *k*-mer were three or more of A or T, the *k*-mer was removed.

5. When a *k*-mer contained more than three times of dinucleotide repeats (e.g., ATATAT and CGCGCG), the *k*-mer was removed.

6. When the GC content of full-length *k*-mer (30 mers) was less than 11 and greater than 17, the *k*-mer was removed.

7. When the GC content of the former and latter half of *k*-mer (15 mers) was less than four and greater than 10, the *k*-mer was removed.

8. When the Tm value of *k*-mer was less than 67 and greater than 82, the *k*-mer was removed.

9. When a *k*-mer matched another *k*-mer within the species and a *k*-mer of another species, the *k*-mer was removed.

After Revision (line 158, Revised MS (clean copy))

Primer specificity for species-specific detection:

1. If a *k*-mer matched another *k*-mer within the same species or a *k*-mer of another species, the *k*-mer was removed.

Tm (primer melting temperature):

2. If the Tm value of a *k*-mer was less than 67 or greater than 82, the *k*-mer was removed.

GC Content: Preferably in the range of 40%–60%. Include 1–2 G or C bases at the 3' end to improve binding stability; however, avoid excessive GC content at the 3' terminus to prevent non-specific binding.

3. If the GC content of a full-length *k*-mer (30-mers) was less than 11 or greater than 17, the *k*-mer was removed.

4. If the GC content of the former or latter half of a *k*-mer (15-mers) was less than four or greater than 10, the *k*-mer was removed.

5. If both the 5' and 3' ends of a *k*-mer were A or T, the *k*-mer was removed.

6. If both the 5' and 3' ends of a *k*-mer contained three or more of G or C, the *k*-mer was removed.

7. If both the 5' and 3' ends of a *k*-mer contained three or more of A or T, the *k*-mer was removed.

Avoid Repeats and Runs: Prevent intra-primer and inter-primer complementarity to reduce primer-dimer formation.

8. If a *k*-mer contained four or more runs of a single base (e.g., AAAAA or GGGG), the *k*-mer was removed.

9. If a *k*-mer contained more than three times of dinucleotide repeats (e.g., ATATAT or CGCGCG), the *k*-mer was removed.

-------------------------------------------------------------------------------------------------------------------------------------------

Comments 4-d

d. In the Filtering *k*-mers section, it seems a bit roundabout to use bowtie and blastn to filter *k*-mers for similarity to the contigs. Under the hood this can probably also be phrased as a *k*-mer matching criterion. I’m curious to know whether you really need bowtie and blast to get a well filtered set or whether by tweaking the parameters you can get the same level of filtering by just using one of these programs.

Response 4-d

We appreciate your suggestion to consider using a single program with adjusted parameters. However, we have concluded that employing both tools is necessary to achieve an optimal balance between computational efficiency and matching accuracy. Bowtie provides a significant advantage in terms of computational speed, enabling us to rapidly filter *k*-mers with a reduced computational burden. However, the accuracy of *k*-mer matching is substantially higher with blastn. By first applying bowtie for initial filtering and subsequently refining the reduced set of *k*-mers using blastn, we are able to maximize both performance and precision in our filtering process. These revisions have been added to the Materials & Methods section. Please refer to line 180.

Added text (line 180, Revised MS (clean copy))

The filtering of unique *k*-mers, which serve as candidates for species-specific primers, was performed using both Bowtie2 [54] and BLASTN [55]. Bowtie2 offers a significant advantage in computational speed, allowing for rapid initial filtering. In contrast, BLASTN permits more mismatches and supports finer matching criteria, providing greater flexibility and accuracy in sequence comparison. By first applying Bowtie2 for preliminary filtering and then refining the reduced set of *k*-mers with BLASTN, we optimize both computational performance and precision in the filtering process.

-------------------------------------------------------------------------------------------------------------------------------------------

Comments 5

5. It is unclear how the paired primer is designed ‘manually from the alignments’. Please elaborate on what selection criteria were used (upstream/downstream from candidate; distance from candidate; similarity/differentiation between species; uniqueness in transcriptome; primer length, etc.). Why were not simply pairs of species-specific *k*-mers occurring on the same contig selected (with potentially additional selection criteria)?

Response 5

Due to the stringent filtering criteria applied to *k*-mers, it was often not feasible to identify primer pairs directly from simple combinations of species-specific *k*-mers located on the same contig. In cases where suitable pairs were identified, the predicted amplicon size was frequently too large for efficient PCR amplification. Moreover, computationally identified species-specific *k*-mers often exhibited unintended sequence matches to other species during sequence alignment check, reducing their specificity. For these reasons, paired primers were designed manually by evaluating alignments to ensure appropriate product sizes, sufficient sequence specificity, and optimal primer properties. Selection criteria included maintaining an appropriate distance for amplification and ensuring primer uniqueness within the transcriptome to minimize cross-amplification. This approach allowed for the practical refinement of primer design beyond automated *k*-mer pairing. These details have been added to the Materials & Methods section. Please refer to line 204.

Added text (line 204, Revised MS (clean copy))

Selection criteria included maintaining an appropriate distance for amplification and ensuring primer specificity within the transcriptome to minimize cross-amplification. This approach enabled the practical refinement of primer design beyond automated *k*-mer pairing.

-------------------------------------------------------------------------------------------------------------------------------------------

Comments 6

6. Potentially related to the point above: it is unclear how the primer pairs for experimental testing were selected from the tens, hundreds or thousands of candidate targets per species. Were all candidates for which a paired target could be identified selected? Or only one or two per species? If the latter, how were those selected?

Response 6

Primer pairs were designed by manually examining the alignment files to identify positions corresponding to species-specific sequences. The alignments were checked to ensure the candidate primer’s specificity across the tested species. Primer pairs were selected for experimental validation based on these positions, and iterative rounds of design and testing were performed until a suitable species-specific primer pair was identified. For most species, a species-specific primer pair was successfully determined after several design and testing cycles. However, despite multiple attempts, we were unable to identify primer pairs capable of species-specific amplification for *T*. *urticae* and *P*. *citri*. This limitation was likely due to the close genetic relationships between these species and their respective relatives, which limited the availability of truly species-specific sequences. We will further clarify these points in the manuscript to address the selection process more explicitly. These points have been clarified in the Results section to explicitly address the primer selection process. Please refer to line 259.

Added text (line 259, Revised MS (clean copy))

For most species, a species-specific primer pair was successfully developed after multiple cycles of design and testing. However, despite multiple attempts, the specific primer pairs designed for *P*. *citri* and the green form of *T*. *urticae* failed to amplify the target species specifically.

-------------------------------------------------------------------------------------------------------------------------------------------

Comments 7

7. What would be the effect of within-species variation? Here, 100-200 individuals are pooled for RNA extraction. How would the contig assembly and *k*-mer counts be affected by genetic variants within these individuals? (Also, related to point 3, this would probably be a benefit of using RNA data over DNA data, exactly because transcribed regions are more constrained in accumulating variation). Please discuss.

Response 7

We agree that RNA-seq data obtained from pooled RNA extractions may exhibit intraspecific variation, which could potentially affect contig assembly and *k*-mer counts. However, due to the small size of individual spider mites, it is challenging to extract sufficient RNA from a single individual to generate high-quality sequencing data. Nonetheless, the de novo assembly statistics from our study, including N50 and maximum contig lengths, are comparable to those reported in other spider mite studies, indicating that the quality of our assembly is suitable for *k*-mer analysis. We also acknowledge that using RNA data instead of DNA data offers an advantage in this context, as transcribed regions are more constrained in their variation, potentially reducing the impact of intraspecific variation on the analyses. These points have been discussed in the Results section. Please refer to line 242.

Added text (line 242, Revised MS (clean copy))

RNA-Seq data were obtained using RNA extracted from 100–200 adult females of each spider mite species. Pooling individuals can introduce intraspecific variation, which could potentially affect the accuracy of downstream analyses. However, as most RNA-Seq data are derived from transcribed regions, they are less affected by noise from intraspecific variation compared to whole-genome DNA-Seq data, which include non-coding or non-transcribed regions. This makes RNA-Seq data particularly advantageous for detecting species-specific markers. The *de* *novo* assembly of the RNA-Seq data used in this study yielded an N50 length of 1,369–2,256 bp and a maximum contig length of 11,286–24,030 bp. These values are comparable to those reported in other spider mite studies [29, 57]. Based on this level of assembly quality, we determined that the data were appropriate for robust *k*-mer analysis.

-------------------------------------------------------------------------------------------------------------------------------------------

Comments 8

8. With about 1200 species in the Tetranychidae family there are many species not represented among the set of primers designed in this study. What do we expect to happen if applying this method to species not included in the design? Will none of the primer pairs amplify? Or only one pair of the closest related species? And if so, would the product have the same length or different? And then, would there be any way to tell this unrepresented species from the target species? Or would multiple primer pairs amplify? What are the implications for interpreting the data?

Response 8

We acknowledge that species not included in this study may produce variable PCR results. In our study, the specific primer pairs designed for *P.* *citri* and the green form of *T*. *urticae* failed to amplify the target species specifically. However, species-specific primers were successfully designed and experimentally validated for *T*. *kanzawai* and its close relative *T*. *parakanzawai*, as well as for *O*. *gotohi* and its close relative *O*. *amiensis*. These points have been added to the Discussion section. Please refer to line 299.

Before Revision

Better methods for designing primers for closely related species are needed.

After Revision (line 299, Revised MS (clean copy))

In contrast, species-specific primers were successfully designed and experimentally validated for *T*. *kanzawai* and its close relative *T*. *parakanzawai*, as well as for *O*. *gotohi* and *O*. *amiensis*. PCR results for closely related species not tested in this study could differ from the expected outcomes. This limitation is not unique to our method; it also affects other molecular diagnostic techniques, such as PCR-RFLP and real-time PCR, which face challenges in differentiating closely related species. Further studies involving a broader range of species are necessary to improve the reliability and practicality of these approaches for comprehensive species identification.

-------------------------------------------------------------------------------------------------------------------------------------------

Comments 9

9. Is there a way to build in a positive control for this design? It seems relevant that when the diagnosis is based on the absence of a PCR band (diagnosis being ‘this is not the target species’) there is a way to check that the PCR actually worked.

Response 9

We confirm that the DNA used in our PCR reactions was tested and successfully amplified using a 28S rDNA primer set, verifying that the extracted DNA was of sufficient quality for amplification. This information has been included in the Materials & Methods section to clarify that a positive control was implemented. Please refer to line 214.

Added text (line 214, Revised MS (clean copy))

To ensure the quality of the extracted DNA and to provide a positive control for the PCR reactions, we tested the DNA using primer sets known to amplify spider mite DNA. Specifically, we targeted the cytochrome c oxidase subunit I (COI) gene of mitochondrial DNA and the 28S nuclear ribosomal RNA (rRNA) gene [45]. The successful amplification of these markers confirms that the extracted DNA is of sufficient quality for reliable PCR analysis.

-------------------------------------------------------------------------------------------------------------------------------------------

Comments 10

10. Would the authors offer a hypothesis as to why some primer pairs fail to amplify anything at all?

Response 10

Several factors may contribute to this outcome. One possible explanation is the presence of sequence mismatches at the primer binding sites caused by genetic variation within or between populations, which may lead to inefficient or failed primer annealing. Additionally, secondary structures within the template DNA could hinder primer binding or polymerase extension. Another contributing factor might be suboptimal primer design parameters, such as melting temperature or GC content, despite careful optimization. A discussion of these potential causes has been added to the Discussion section. Please refer to line 307.

Added text (line 307, Revised MS (clean copy))

Of the 45 primer pairs designed in this study, 10 did not amplify any products (S2 Table). Several factors may have contributed to this outcome. One possible explanation is the presence of sequence mismatches at the primer binding sites, caused by genetic variation within or between populations, which may lead to inefficient or failed primer annealing. Additionally, secondary structures within the template DNA may hinder primer binding or polymerase extension. Another contributing factor may be suboptimal primer design parameters, such as melting temperature or GC content, despite careful optimization. To enhance the robustness of primer sets, their efficiency should be tested across a broader range of populations and closely related species.
