# Supplementary material for: A PCR primer design method for identifying spider mite species using k-mer counting
Source: PLoS One. 2025 Jun 9;20(6):e0321199. doi: 10.1371/journal.pone.0321199 (PMC12148119; doi:10.1371/journal.pone.0321199)
Supplement: Attachment — Submitted filename: Response to Reviewers 20250217.docx [file pone.0321199.s010.docx]

Dear Reviewers,

We sincerely thank you for your thorough review and constructive comments on our manuscript. Your valuable suggestions have greatly contributed to improving the quality and clarity of our work.

In this document, we have provided detailed responses to each of your comments. We have carefully addressed all points and revised the manuscript accordingly. Below, we outline our responses to your comments, with changes in the manuscript highlighted in the revised version.

Sincerely,

Tomoko Matsuda

Nihon BioData Corporation

-------------------------------------------------------------------------------------------------------------------------------------------

Reviewer #2's comments 1

I would remove "the" from the title, so:

A PCR primer design method for identifying spider mite species using the *k*-mer counting

Response 1

In the manuscript, we had already removed 'the'; however, the title entered in the submission form still included it. The manuscript already contained the correct title, so we have now updated the title in the submission form accordingly.

-------------------------------------------------------------------------------------------------------------------------------------------

Reviewer #2's comments 2

Re comment 5: did you recycle any of the methods from the initial candidate primer identification steps to check the specificity/uniqueness of the complementary primer?

Response 2

We appreciate your suggestion and have conducted an additional BLASTN search to further verify the specificity and uniqueness of the complementary primer. This step has been incorporated into the manuscript.

Added text (line 210, Revised MS (clean copy))

To confirm the uniqueness of the species-specific primers, we conducted additional BLASTN searches to compare the species-specific primers with the contigs from the 19 assemblies after clustering with CD-HIT.

-------------------------------------------------------------------------------------------------------------------------------------------

Reviewer #2's comments 3

Re comment 6: are there any heuristics for which primers pairs to pick first or was that completely at random? And then select another pair at random if the first pair failed?

All other comments have been satisfactorily addressed.

Response 3

The primer pairs were primarily designed at random, including cases where the initial primer failed. However, since alignments were checked when designing the primer pairs, if an alignment was particularly suitable for primer design, additional primers were sometimes designed from the same contig. As a result, there are cases where primers were designed from the same transcript, such as XM_015937235.2 (tetur01g00100) and NW_015449938.1 (tetur09g06570) in S2 Table. We have also incorporated this clarification into the manuscript.

Added text (line 259, Revised MS (clean copy))

The primer pairs were initially designed randomly. However, since sequence alignments were carefully examined during the primer design process, additional primers were occasionally designed from the same contig when a particular alignment was deemed highly suitable for primer design. As a result, multiple primers were designed from the same transcript in certain cases, such as XM_015937235.2 and NW_015449938.1, as shown in Table 2 and S2 Table. Finally, 43 PCR primers were designed to target species-specific sequences.
